# Global, regional, and national HIV/AIDS disease burden levels and trends in 1990–2019: A systematic analysis for the global burden of disease 2019 study

**DOI:** 10.3389/fpubh.2023.1068664

**Published:** 2023-02-15

**Authors:** Xuebin Tian, Jingjing Chen, Xi Wang, Yiwen Xie, Xiaodi Zhang, Dating Han, Haijing Fu, Wanpeng Yin, Nanping Wu

**Affiliations:** ^1^State Key Laboratory for Diagnosis and Treatment of Infectious Diseases, National Clinical Research Center for Infectious Diseases, National Medical Center for Infectious Diseases, Collaborative Innovation Center for Diagnosis and Treatment of Infectious Diseases, The First Affiliated Hospital, Zhejiang University School of Medicine, Hangzhou, Zhejiang, China; ^2^Jinan Microecological Biomedicine Shandong Laboratory, Jinan, Shandong, China; ^3^Shandong Second Provincial General Hospital, Jinan, Shandong, China; ^4^Center for Genomic and Personalized Medicine, Guangxi Medical University, Nanning, China

**Keywords:** HIV/AIDS, burden of disease, prevalence, deaths, disability-adjusted life years, trend, risk factors

## Abstract

**Background:**

Since the first HIV/AIDS case appeared in 1980s, HIV/AIDS has been the focus of international attention. As a major public health problem, there are epidemiological uncertainties about the future of HIV/AIDS. It is important to monitor the global statistics of HIV/AIDS prevalence, deaths, disability adjusted life years (DALYs), and risk factors for adequate prevention and control.

**Methods:**

The Global Burden of Disease Study 2019 database was used to analyze the burden of HIV/AIDS in 1990–2019. By extracting global, regional, and national data on HIV/AIDS prevalence, deaths, and DALYs, we described the distribution by age and sex, explored the risk factors, and analyzed the trends in HIV/AIDS.

**Results:**

In 2019, there were 36.85 million HIV/AIDS cases (95% UI: 35.15–38.86 million), 863.84 thousand deaths (95% UI: 78.61–99.60 thousand), and 47.63 million (95% UI: 42.63–55.65 million) DALYs. The global age-standardized HIV/AIDS prevalence, death, and DALY rates were 454.32 (95% UI: 433.76–478.59), 10.72 (95% UI: 9.70–12.39), and 601.49 (95% UI: 536.16–703.92) per 100,000 cases, respectively. In 2019, the global age-standardized HIV/AIDS prevalence, death, and DALY rates increased by 307.26 (95% UI: 304.45–312.63), 4.34 (95% UI: 3.78–4.90), and 221.91 (95% UI: 204.36–239.47) per 100,000 cases, respectively, compared to 1990. Age-standardized prevalence, death, and DALY rates decreased in high sociodemographic index (SDI) areas. High age-standardized rates were observed in low sociodemographic index areas, while low age-standardized rates were observed in high sociodemographic index areas. In 2019, the high age-standardized prevalence, death, and DALY rates were predominant in Southern Sub-Saharan Africa, and global DALYs peaked in 2004 and subsequently decreased. The highest global HIV/AIDS DALYs were in the 40–44 age group. The main risk factors affecting HIV/AIDS DALY rates included behavioral risks, drug use, partner violence, and unsafe sex.

**Conclusions:**

HIV/AIDS disease burden and risk factors vary by region, sex, and age. As access to health care increases across countries and treatment for HIV/AIDS infection improves, the HIV/AIDS disease burden is concentrated in areas with low SDIs, particularly in South Africa. Regional differences should be fully considered to target optimal prevention strategies and treatment options based on risk factors.

## 1. Introduction

HIV/AIDS prevention and control is a major global health challenge (1). Due to the joint efforts of countries around the world, developed countries have made progress in curbing the HIV/AIDS epidemic. However, in several developing and resource-limited countries, HIV/AIDS continues to affect and take human lives (2). In the past 30 years, HIV/AIDS has spread at an alarming rate and is among the top 10 global burden of diseases (2). The Joint United Nations Program on HIV/AIDS (UNAIDS) reported that 38 million (31.6–44.5 million) people were living with HIV in 2019, of which 36.2 million (30.2–42.5 million) were adults and 1.8 million (1.3–2.2 million) were children (0–14 years), with ~1.7 million people newly infected with HIV/AIDS and 690,000 people dying from HIV/AIDS-related diseases (3).

HIV/AIDS affects human development. As the life expectancy of people living with HIV/AIDS continues to increase and the mortality rate continues to decrease, many people living with HIV/AIDS are in a state of disease. HIV/AIDS treatment access is not universal, and the prospect of treatments and effective vaccines is uncertain (4). Advances in medical technology have increased both the life expectancy of HIV/AIDS patients and the burden on families and societies. In addition, the weakening of the immune system due to HIV/AIDS often leads to the co-morbidity of other diseases (e.g., tuberculosis), the treatment of which can lead to an increased burden of HIV/AIDS. Therefore, prevention and awareness represent feasible approaches.

While most studies have focused on HIV/AIDS complications and antiretroviral therapies (5–7), few studies have reported the global burden and risk factors due to HIV/AIDS. In this study, we study used data from the Global Burden of Disease 2019 study to analyze the prevalence, deaths, disability-adjusted life years (DALYs), and risk factors by age and sex from 1990 to 2019. Such information is vital to policy development, implementation, and resource allocation (8).

## 2. Methods

### 2.1. Data source

We obtained data on HIV/AIDS prevalence, deaths, years of life lost (YLLs), years lived with disability (YLDs), DALYs, and risk factors for 1990–2019 from the 2019 Global Burden of Disease Study data (GBD 2019), which presents prevalence, death, DALY rates per 100,000 people and 95% uncertainty intervals (UIs) (9). The data can be downloaded from the Global Health Data Exchange database at the University of Washington Institute for Health Metrics and Evaluation (IHME; http://ghdx.healthdata.org/gbd-results-tool).

GBD 2019 constitutes a systematic analysis. Data was collected through censuses, household surveys, vital statistics, air pollution monitors, civil registration, disease registries, health care usage, satellite imaging, disease alerts, and other sources. The Cause of Death Ensemble model and spatiotemporal Gaussian process regression were used to determine cause-specific death rates and cause fractions (10). GBD 2019 quantifies the extent of health loss due to disease, injury, and risk factors by age, sex, and geographic location at a given point in time, using indicators such as prevalence, deaths, YLD, YLL, and DALYs (10–12). DALYs, which is equal to YLD + YLL, was proposed by WHO epidemiologists Alan Lopez and Christopher Murray of the Harvard School of Public Health as a novel indicator of disease burden and is included as a routine indicator in GBD studies (13, 14). We calculated age-standardized rates in GBD 2019 using the GBD World Population Age Standard (3). Annual percentage changes in GBD 2019 reflect trends in age-standardized rates over a certain interval. For example, when the estimated annual percentage change is equal to 1, it means that the age-standardized rates increase at a rate of 1% per year during the observation period. The estimated annual percentage change >0 and its 95% UI > 0 denoted an increasing age-standardized rate trend. By contrast, a decreasing age-standardized rate trend was identified when the estimated annual percentage change estimation < 0 and its 95% UI did not exceed 0. The studies included in the modeling process can be found here https://ghdx.healthdata.org/gbd-2019/data-input-sources (9, 15).

### 2.2. Statistical analyses

GBD 2019 provides an accurate and comprehensive summary of the global disease burden for 369 diseases and injuries for 1990–2019 by age and sex and systematically analyzes the burden of disease due to 87 risk factors for comparison between different countries and regions (9). To compare the variability among different regions and ages and between sexes, we obtained HIV/AIDS disease burden data. We divided the data into five categories: (1) sociodemographic index (SDI), (2) 21 regions, (3) 204 countries, (4) age (e.g., five age categories), and (5) sex. We used GraphPad Prism 8.0 (GraphPad Software Inc., San Diego, CA, USA) to generate charts and used R version 4.2.1 [R Core Team (2021). R: A language and environment for statistical computing. R Foundation for Statistical Computing, Vienna, Austria. https://www.R-project.org/] for data analyses.

## 3. Results

### 3.1. Global level

**Figure 1:**
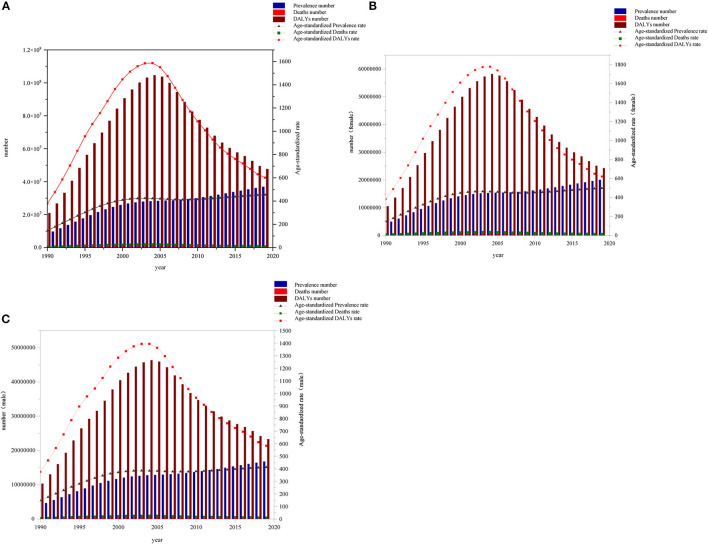
Global **(A)**, female **(B)**, and male **(C)** prevalence cases, deaths, DALYs and age-standardized prevalence rate, death rate, DALY rate due to HIV/AIDS from 1990 to 2019.

The number and age-standardized prevalence of HIV/AIDS increased globally from 1990 to 2019, rising rapidly between 1990 and 2000, and slowing down from 2000 to 2019. From 1990 to 2019, the global number of deaths and age-standardized death rates increased slowly until 2005 and subsequently had a downward trend. Global DALYs and age-standardized DALY rates increased rapidly from 1990 to 2005, peaked in 2005, and declined from 2005 to 2019 (Figure 1).

In 2019, there were 3.69 million (95% UI: 3.52–3.89 million) HIV/AIDS cases worldwide with a prevalence rate of 476.23 (95% UI: 454.27–502.19) per 100,000 cases and an age-standardized rate of 454.32 (95% UI: 433.76–478.59) per 100,000 cases. In 2019, we observed an increase of 307.27 (95% UI: 304.45–312.63) per 100,000 cases compared with 1990 and 863,800 deaths (95% UI: 99.60–786,100). The DALYs were 4,763.22 (95% UI: 4,263.10–5,565.00). The death rate was 11.16 (95% UI: 10.16–12.87) per 100,000 cases, and the age-standardized rate was 10.72 (95% UI: 9.70–12.39) per 100,000 cases. Age-standardized rates increased by 4.34 (95% UI: 3.77–4.89) per 100,000 cases compared with 1990. The global DALY rate in 2019 was 615.60 (95% UI: 550.97–719.23) per 100,000 cases and the age-standardized rate was 601.49 (95% UI: 536.16–703.92) per 100,000 cases, compared with 1990, an increase of 221.91 (95% UI: 204.36–239.47) per 100,000 cases (Tables 1, 2).

**Table 1:** Prevalence cases, deaths, and disability-adjusted life years (DALYs) for HIV/AIDS in 2019 and percentage change in age standardized rates (ASRs) per 100,000, by SID and global burden of disease region, from 1990 to 2019.

	**Prevalence (95%UI)**			**Deaths (95%UI)**			**DALYs (95%UI)**		
	**Number (95% UI)**	**Age-standardized rate (95% UI)**	**Percentage change in ASRs from 1990 to 2019**	**Number (95% UI)**	**Age-standardized rate (95% UI)**	**Percentage change in ASRs from 1990 to 2019**	**Number (95% UI)**	**Age-standardized rate (95% UI)**	**Percentage change in ASRs from 1990 to 2019**
High SDI	2,373,033.44 (1,348,018.47–3,395,221.18)	183.72 (105.10-265.36)	0.44 (0.22–0.64)	10,626.48 (10,119.63–11,749.01)	0.84 (0.80–0.96)	−0.79 (−0.80 to −0.76)	620,844.22 (521,797.60–747,414.65)	51.78 (43.65–62.36)	−0.76 (−0.79 to −0.71)
High-middle SDI	2,767,164.63 (2,417,462.40–3,248,217.43)	165.78 (143.15–196.55)	4.04 (3.40–4.70)	50.804.76 (49,108.70–53,139.36)	3.04 (2.93–3.20)	1.07 (0.95–1.20)	2,752,778.76 (2,618,336.76–2,938,583.91)	172.44 (163.60–184.18)	1.07 (0.94–1.21)
Low SDI	10,216,979.58 (9,686,277.59–10,786,450.61)	1,154.52 (1,098.27–1,218.70)	0.22 (0.03–0.46)	267,800.41 (233,312.82–324,054.24)	30.11 (27.06–35.09)	−0.33 (−0.48 to −0.07)	15,551,583.36 (13,229,664.41–18,952,911.09)	568.70 (510.39–672.63)	−0.37 (−0.49 to −0.16)
Low-middle SDI	8,962,247.69 (8,523,022.19–9,497,671.37)	517.17 (491.86–548.27)	2.31 (1.53–3.23)	255,788.36 (228,673.13–295,039.43)	14.93 (13.52–17.01)	1.67 (0.81–2.85)	13,950,233.97 (12,220,876.20–16,341,379.90)	795.43 (703.30–921.08)	1.37 (0.74–2.16)
Middle SDI	12,501,850.83 (11,868,369.48–13,326,058.38)	472.33 (447.99–503.74)	13.86 (12.07–16.08)	278,034.41 (252,272.28–325,774.93)	10.53 (9.53–12.40)	9.12 (7.74–11.08)	14,714,395.91 (13,273,529.47–17,332,747.71)	1,578.46 (1,387.12–1,877.83)	8.05 (7.10–9.32)
Global	36,848,153.96 (35,149,001.88–38,856,666.01)	454.32 (433.76–478.59)	2.09 (1.75–2.52)	863,837.35 (786,074.86–996,044.87)	10.72 (9.70–12.39)	0.68 (0.41–1.09)	47,632,184.17 (42,630,989.91–55,650,039.40)	601.49 (536.16–703.92)	0.58 (0.37–0.89)
Southern Sub-Saharan Africa	10,292,824.75 (9,799,857.32–10,790,807.29)	13,291.17 (12,661.43–13,924.75)	11.26 (5.78–21.72)	188,071.84 (164,259.75–227,046.14)	247.28 (220.41–291.71)	6.36 (2.78–12.51)	10,110,566.81 (8,689,805.41–12,327,661.51)	12,776.70 (11,185.64–1,529,806)	5.12 (2.3–39.88)
Eastern Sub-Saharan Africa	10,452,876.58 (10,976,270.2–49,962,642.30)	3,422.91 (3,268.72–3,583.83)	0.32 (0.15–0.55)	246,385.41 (217,705.21–293,243.32)	80.12 (73.44–90.78)	−0.33 (−0.49 to −0.03)	14,398,355.18 (12,394,140.52–17,302,740.07)	4,141.16 (3,732.24–4,808.22)	−0.37 (−0.50 to −0.15)
Western Sub-Saharan Africa	4,192,276.04 (3,945,736.32–4,464,333.01)	1,248.18 (1,339.6–41,166.93)	1.22 (0.47–2.15)	165,003.45 (138,977.90–198,994.01)	50.66 (44.70–58.94)	1.22 (0.47–2.18)	8,861,100.44 (7,347,940.50–10,890,249.43)	2,460.72 (2,128.06–2,914.02)	0.95 (0.32–1.71)
Central Sub-Saharan Africa	1,095,816.09 (943,016.06–1,261,687.32)	1,084.37 (933.09–1,247.23)	0.01 (−0.25–0.38)	40,093.82 (32,821.29–50,509.94)	40.90 (34.54–49.44)	−0.22 (−0.43–0.10)	2,221,933.82 (1,777,106.88–2,839,562.18)	2,050.21 (1,695.81–2,546.25)	−0.28 (−0.46 to −0.02)
Oceania	97,264.64 (4,227.70–280,661.72)	804.07 (35.13–2,301.26)	89.93 (5.35–277.17)	4,175.15 (1,348.30–11,312.00)	35.61 (12.45–96.39)	112.06 (23.59–439.46)	221,642.57 (62,165.85–607,020.87)	1,775.74 (535.12–4,894.80)	87.54 (19.14–317.34)
Caribbean	318,289.96 (275,171.60–367,448.48)	647.06 (558.26–748.28)	0.97 (0.27–2.10)	9,374.20 (7,889.29–11,451.78)	19.03 (15.97–23.32)	0.27 (−0.13–0.73)	484,655.62 (403,462.58–602,610.54)	1,002.14 (828.70–1,249.77)	0.16 (−0.20–0.62)
Eastern Europe	1,436,465.47 (1,175,115.22–1,763,653.54)	613.42 (493.02–765.06)	18.33 (13.09–24.17)	25,993.85 (25,634.80–26,349.73)	10.94 (10.80–11.09)	4.08 (4.00–4.17)	1,453,867.59 (1,399,239.51–1,525,790.51)	630.46 (606.93–661.93)	4.25 (4.11–4.43)
Andean Latin America	139,107.16 (105,854.99–191,142.11)	215.10 (166.36–294.83)	3.49 (1.48–5.33)	4,340.44 (2,815.65–8,265.14)	6.877 (4.38–12.97)	1.94 (0.32–4.68)	259,655.39 (163,342.69–492,514.62)	401.96 (251.36–762.66)	2.14 (0.46–5.09)
Tropical Latin America	907,188.14 (779,713.16–1,064,492.28)	362.77 (310.53–425.98)	2.30 (1.39–3.33)	16,131.13 (15,784.36–16,504.57)	6.47 (6.32–6.62)	0.17 (0.14–0.20)	840,174.92 (802,376.82–885,859.90)	342.53 (326.79–361.83)	0.08 (0.03–0.13)
Southeast Asia	1,715,246.22 (147,688,276–2,125,087.58)	237.53 (205.49–293.09)	7.38 (6.21–9.48)	42,950.70 (35,861.06–54,416.12)	6.00 (4.96–7.70)	11.25 (8.33–15.43)	2,392,333.53 (2,057,902.86–2,957,693.74)	336.51 (288.07–419.04)	9.36 (7.58–11.61)
Central Latin America	516,354.51 (420,650.09–629,394.03)	198.7 (163.10–240.67)	3.81 (3.11–4.86)	12,021.69 (11,456.65–12,517.18)	4.66 (4.43–4.85)	0.66 (0.58–0.73)	632,107.84 (605,348.50–661,065.59)	244.00 (233.50–255.72)	0.69 (0.62–0.76)
Southern Latin America	256,071.78 (150,564.11–382,254.77)	357.22 (206.14–538.55)	3.09 (2.35–3.90)	2,446.49 (2,405.58–2,486.62)	3.35 (3.29–3.40)	1.26 (1.21–1.31)	138,078.33 (123,608.73–162,496.72)	192.41 (171.92–226.98)	1.34 (1.16–1.65)
South Asia	1,937,497.17 (1,691,087.52–2,394,805.67)	107.18 (93.46–131.09)	15.71 (8.38–27.47)	52,070.06 (42,176.16–82,536.00)	2.89 (2.35–4.64)	24.02 (10.64–47.67)	2,927,119.87 (2,331,734.87–4,676,766.64)	160.06 (128.20–254.57)	17.21 (9.47–28.73)
High-income North America	1,835,630.31 (982,211.80–2,704,937.11)	408.70 (219.92–605.67)	0.34 (0.10–0.57)	7,308.04 (7,225.3–7,388.69)	1.64 (1.62–1.66)	−0.82 (−0.82 to −0.82)	433,184.12 (62,145.53–530,234.96)	102.00 (85.78–123.58)	−0.79 (−0.82 to −0.75)
East Asia	570,193.73 (294,164.20–1,082,600.98)	31.73 (16.54–59.78)	4.69 (2.38–7.48)	32,678.11 (26,106.32–39,649.85)	1.74 (1.40–2.09)	6.37 (3.35–29.37)	1,441,746.08 (1,136,935.93–1,811,660.06)	82.37 (65.91–102.64)	5.36 (3.00–18.74)
North Africa and Middle East	203,878.67 (106,281.69–407,533.70)	31.90 (16.81–62.46)	4.64 (1.07–12.98)	9,433.33 (5,505.64–18,554.68)	1.51 (0.88–3.04)	5.38 (1.53–13.91)	509,127.61 (292,583.54–1,024,989.37)	79.29 (45.26–160.62)	4.88 (1.31–12.45)
Central Asia	63,238.60 (52,525.12–80,385.06)	62.87 (52.31–78.72)	5.85 (3.91–8.49)	1,275.04 (1,228.94–1,325.34)	1.28 (1.23–1.33)	1.39 (1.26–1.52)	71,148.96 (67,777.39–74,763.21)	70.86 (67.48–74.47)	1.43 (1.30–1.57)
Central Europe	39,963.25 (32,196.87–54,552.83)	31.22 (24.77–42.95)	5.95 (4.37–8.20)	446.58 (423.96–502.38)	0.35 (0.34–0.39)	−0.12 (−0.19 to 0.03)	24,404.90 (22,478.46–28,326.11)	21.11 (19.48–23.99)	−0.19 (−0.27 to −0.05)
Australasia	19,867.54 (13,195.49–27,002.97)	55.71 (35.99–77.60)	0.22 (0.03–0.40)	79.35 (77.07–81.52)	0.23 (0.23–0.24)	−0.88 (−0.88 to −0.88)	4,893.66 (4,164.79–5,909.81)	14.80 (12.71–17.84)	−0.85 (−0.87 to −0.83)
High-income Asia Pacific	80,684.35 (47,945.22–115,349.65)	30.87 (18.83–44.05)	6.97 (4.89–13.70)	340.17 (329.62–351.40)	0.13 (0.13–0.14)	0.97 (0.90–1.05)	20,146.30 (15,976.80–25,933.92)	8.82 (7.29–10.97)	1.36 (1.03–1.80)

**Table 2:** Prevalence cases, deaths, and disability-adjusted life years (DALYs) for HIV/AIDS in 1990 per 100,000, by SID and global burden of disease region, from 1990 to 2019.

	**Prevalence (95%UI)**		**Deaths (95%UI)**		**DALYs (95%UI)**	
	**Number (95% UI)**	**Age-standardized rate (95% UI)**	**Number (95% UI)**	**Age-standardized rate (95% UI)**	**Number (95% UI)**	**Age-standardized rate (95% UI)**
High SDI	1,149,170.00 (724,379.78–1,654,718.22)	127.71 (80.28–184.06)	35,979.72 (35,742.48–36,229.30)	3.97 (3.94–4.00)	1,915,003.44 (1,858,589.87–1,987,774.94)	213.70 (207.53–221.73)
High-middle SDI	407,605.98 (331,346.41–501,394.57)	32.90 (26.79–40.53)	17,533.65 (16,953.25–18,488.47)	1.47 (1.42–1.55)	999,437.89 (964,758.42–1,052,394.26)	83.24 (80.29–87.86)
Low SDI	4,111,875.16 (3,456,193.62–4,802,206.46)	945.90 (797.34–1,108.54)	204,761.98 (144,455.32–285,684.63)	45.25 (30.54–64.98)	12,877,091.49 (9,534,039.39–17,312,230.30)	2,501.89 (1,762.13–3,511.47)
Low-middle SDI	1,615,973.94 (1,273,820.18–2,093,361.93)	156.25 (123.03–202.09)	60,609.40 (41,877.30–91,697.87)	5.59 (3.72–8.74)	3,997,997.43 (2,885,369.60–5,771,796.09)	336.16 (235.36–502.83)
Middle SDI	537,066.53 (452,995.78–625,153.71)	31.79 (26.91–37.04)	17,179.86 (13,958.19–21,913.86)	1.04 (0.83–1.34)	1,106,451.28 (942,455.87–1,348,929.71)	62.84 (52.90–77.69)
Global	7,828,845.99 (6,876,622.78–8,848,630.56)	147.06 (129.31–165.96)	336,386.61 (255,683.19–452,193.62)	6.38 (4.81–8.62)	20,915,848.32 (16,525,433.41–27,291,854.64)	379.58 (296.69–499.56)
Southern Sub-Saharan Africa	533,077.97 (285,665.53–959,585.86)	1,084.11 (580.48–1,945.06)	16,850.37 (8,743.71–33,464.21)	33.61 (17.46–68.92)	1,145,025.58 (586,553.19–2,206,818.30)	2,089.37 (1,097.06–4,074.81)
Eastern Sub-Saharan Africa	3,913,184.34 (3,347,508.56–4,461,834.73)	2,592.57 (2,202.86–2,957.04)	187,701.45 (134,136.04–260,736.98)	119.85 (80.37–172.33)	11,966,038.93 (8,965,866.06–16,072,456.32)	6,585.85 (4,635.43–9,198.11)
Western Sub-Saharan Africa	877,632.81 (626,862.44–1,319,941.86)	562.62 (403.78–847.63)	37,028.66 (24,133.76–59,777.29)	22.87 (14.72–37.49)	2,310,936.48 (1,545,403.99–3,678,241.72)	1,259.76 (832.04–2,042.92)
Central Sub-Saharan Africa	482,571.40 (354,178.57–631,746.67)	1,078.34 (792.34–1,433.15)	23,982.24 (16,278.02–34,974.23)	52.59 (35.09–78.01)	1,484,165.51 (1,022,173.82–2,121,911.64)	2,832.80 (1,912.07–4,160.84)
Oceania	481.41 (227.12–1,518.43)	8.84 (4.08–28.42)	21.10 (9.42–68.08)	0.31 (0.11–1.06)	1,531.02 (779.89–4,674.18)	20.06 (8.96–65.33)
Caribbean	113,830.65 (72,992.55–171,668.69)	328.83 (209.82–498.69)	5,082.12 (3,583.57–8,009.61)	15.01 (10.69–23.25)	305,861.79 (211,924.65–484,239.39)	860.36 (602.64–1,359.01)
Eastern Europe	76,841.40 (50,618.18–114,861.22)	31.74 (20.74–47.70)	5,080.68 (5,021.85–5,135.70)	2.15 (2.13–2.17)	275,235.72 (268,880.47–285,500.94)	119.99 (117.32–124.13)
Andean Latin America	16,765.88 (11,159.17–31,044.01)	47.93 (31.60–98.08)	772.17 (557.39–1,529.72)	2.31 (1.62–4.70)	47,231.77 (35,493.10–89,982.23)	127.79 (93.61–253.97)
Tropical Latin America	170,795.98 (119,154.09–232,375.46)	109.84 (77.17–149.11)	8,105.87 (7,968.61–8,236.20)	5.54 (5.45–5.62)	486,694.20 (474,428.50–500,569.68)	317.63 (309.75–326.95)
Southeast Asia	124,263.51 (91,852.28–160,225.86)	28.34 (20.98–37.12)	2,245.32 (1,839.36–2,921.33)	0.49 (0.39–0.67)	158,805.44 (139,250.48–188,776.35)	32.47 (27.94–39.95)
Central Latin America	60,058.77 (41,259.51–82,505.37)	41.34 (28.75–56.85)	3,849.70 (3,810.09–3,890.80)	2.80 (2.77–2.83)	216,180.39 (212,700.55–220,115.90)	144.37 (142.13–146.85)
Southern Latin America	42,970.57 (29,875.37–57,644.51)	87.30 (60.89–116.76)	710.70 (700.28–721.35)	1.48 (1.46–1.50)	39,811.74 (37,860.62–42,204.21)	82.10 (78.15–87.03)
South Asia	60,665.43 (35,117.63–107,972.25)	6.41 (3.84–11.02)	1,430.84 (892.16–2,701.84)	0.12 (0.06–0.26)	116,735.41 (82,665.02–191,821.01)	8.79 (5.74–15.58)
High–income North America	958,825.66 (581,240.38–1,416,377.62)	304.25 (183.08–450.38)	28,592.25 (28,356.24–28,831.32)	9.11 (9.03–9.18)	1,532,150.62 (1,483,196.99–1,595,118.40)	487.94 (472.43–507.80)
East Asia	68,075.80 (50,184.63–89,168.60)	5.57 (4.18–7.50)	2,736.66 (734.19–3,982.89)	0.24 (0.07–0.34)	158,454.72 (54,124.29–219,983.19)	12.95 (4.50–17.97)
North Africa and Middle East	16,387.80 (8,888.46–32,834.86)	5.65 (3.10–11.25)	719.34 (333.67–1,628.21)	0.24 (0.11–0.55)	46,196.71 (22,294.90–108,907.59)	13.48 (6.54–29.64)
Central Asia	6,178.76 (4,086.44–8,645.56)	9.18 (6.21–12.71)	327.22 (313.25–339.51)	0.54 (0.51–0.56)	18,692.40 (17,960.96–19,383.55)	29.21 (28.09–30.31)
Western Europe	282,914.73 (211,574.88–357,178.57)	69.81 (52.06–88.12)	10,119.12 (10,014.85–10,227.32)	2.46 (2.44–2.49)	548,976.91 (536,166.46–562,738.55)	137.10 (133.96–140.46)
Central Europe	5,802.62 (3,871.02–7,995.44)	4.49 (2.96–6.26)	471.52 (433.49–498.82)	0.40 (0.37–0.42)	28,310.42 (25,913.07–29,915.36)	26.01 (23.87–27.47)
Australasia	10,090.83 (7,403.50–12,657.92)	45.73 (33.48–57.41)	430.34 (419.85–441.90)	1.95 (1.91–2.01)	22,078.63 (21,297.25–22,935.33)	100.26 (96.71–104.12)
High–income Asia Pacific	7,429.67 (3,478.72–12,457.49)	3.87 (1.77–6.53)	128.92 (126.07–131.74)	0.07 (0.06–0.07)	6,733.95 (6,133.76–7,427.49)	3.74 (3.43–4.11)

**Figure 2:**
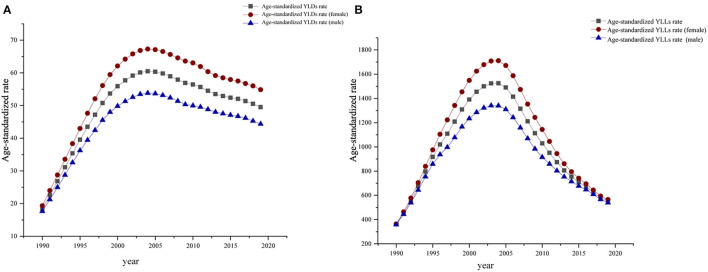
Global age-standardized YLD **(A)** and age-standardized YLL **(B)** from 1990 to 2019.

**Figure 3:**
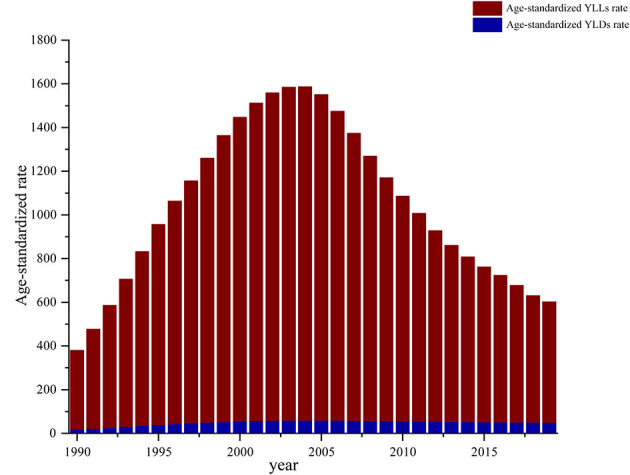
Global age-standardized YLDs rate and age-standardized YLLs rate as a percentage of DALYS, 1990–2019.

From 1990 to 2019, the age-standardized DALY rate, YLD rate, and YLL rate were higher in women with HIV/AIDS than in men with HIV/AIDs, suggesting that the disease burden was greater in women. The global trends of DALYs, YLDs, and YLLs in males and females were basically the same, reaching the highest value in 2005 (Figure 2). The global burden of HIV/AIDS was dominated by death, and the proportion of YLDs in DALYs increased with time (Figure 3).

### 3.2. Regional level

In 2019, low SDI areas had the highest age-standardized prevalence rate, age-standardized death rate, and age-standardized DALY rate. Percentage changes in age-standardized prevalence rates from 1990 to 2019 were the highest in middle SDI areas and lowest in low SDI areas. Percentage changes in age-standardized death rate from 1990 to 2019 were the highest in middle SDI areas and lowest in high SDI areas. Percentage changes in age-standardized DALY rates from 1990 to 2019 were the highest in the middle SDI areas and lowest in high SDI areas (Table 1).

**Figure 4:**
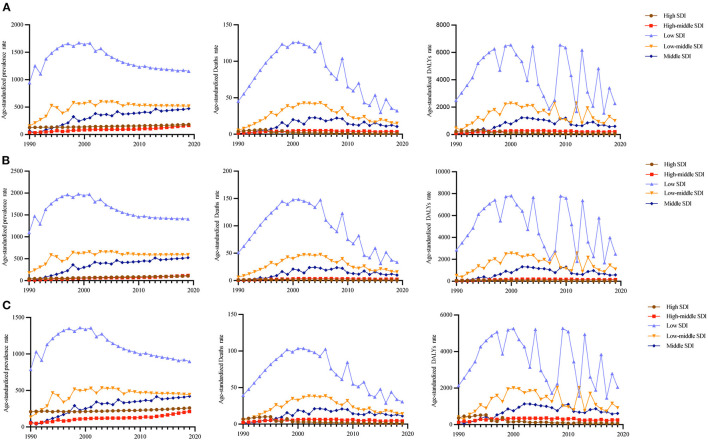
Age-standardized prevalence, death, and DALY rates by both **(A)**, in females **(B)**, and males **(C)** in different SID areas.

From 1990 to 2019, the prevalence of high and high-middle SDI areas increased over time, while deaths and DALYs increased at first and subsequently decreased. The age-standardized prevalence in low SDI areas increased at first and subsequently decreased, reaching a maximum in 2001. The age-standardized death rates fluctuated and decreased, while age-standardized DALYs fluctuated significantly. In low-middle SDI areas, the age-standardized prevalence showed a fluctuating upward trend. Age-standardized deaths first increased and subsequently decreased, and DALYs fluctuated. Age-standardized prevalence, age-standardized deaths, and DALYs increased at first and subsequently decreased in middle SDI areas. The trend among males and females was consistent with the general trend (Figure 4).

In 2019, Southern Sub-Saharan Africa (13,291.18; 95% UI: 12,661.43–13,924.75). and high-income Asia Pacific (30.87; 95% UI: 18.83–44.05) had the highest and lowest age-standardized prevalence rates, respectively. Southern Sub-Saharan Africa (247.28; 95% UI: 220.41–291.71) and high-income Asia Pacific (0.13; 95% UI: 0.13–0.14) had the highest and lowest age-standardized death rates, respectively. Southern Sub-Saharan Africa (12,776.70; 95% UI: 11,185.64–15,298.06) and high-income Asia Pacific (8.82; 95% UI: 7.29–10.97) had the highest and lowest age-standardized DALY rates, respectively (Table 1).

**Figure 5:**
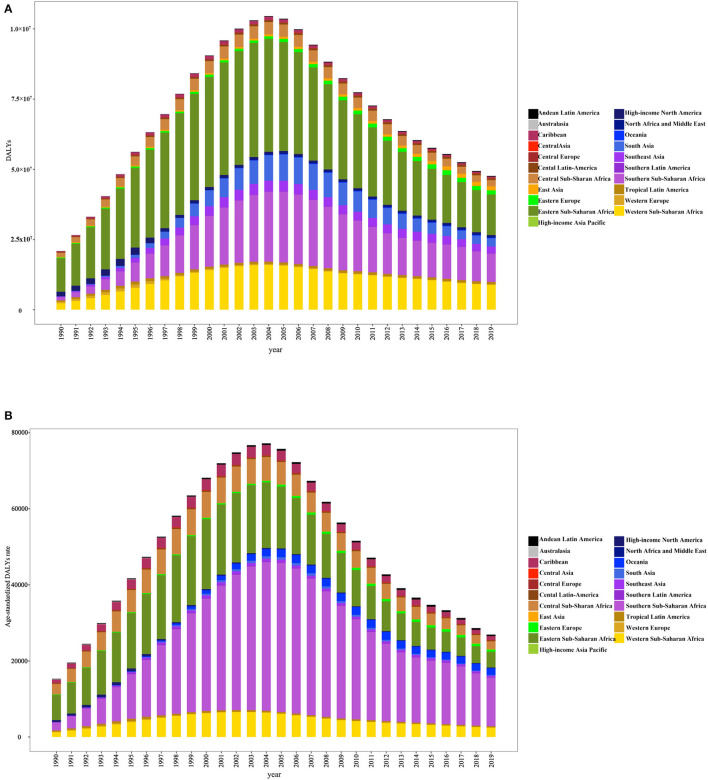
DALYs **(A)** and age-standardized DALY rates **(B)** in 21 disease burden regions from 1990 to 2019.

When comparing regional differences in disease burden across 21 regions, we observed that the disease burden was higher in Eastern sub-Saharan Africa, South Asia, Southern sub-Saharan Africa, and Western sub-Saharan Africa (Figure 5A), especially in Eastern and Southern sub-Saharan Africa (Figure 5).

### 3.3. National level

**Figure 6:**
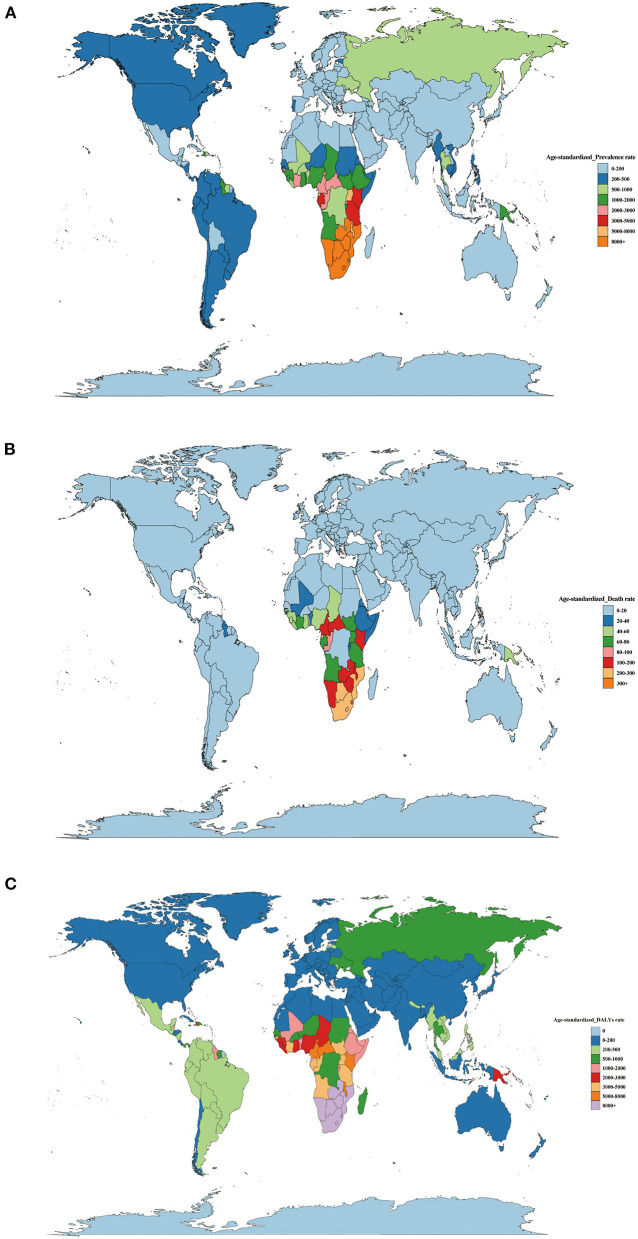
**(A)** Age-standard prevalence rates, **(B)** age-standard death rates, **(C)** age-standard DALY rates of HIV/AIDS per 100,000 cases in 2019 by country.

There are national differences in the disease burden of HIV/AIDS. Africa had the highest disease burden of HIV/AIDS. In 2019, out of 204 countries, Eswatini had the highest age-standardized prevalence (22,031.68; 20,432.73–23,878.50), followed by Lesotho (19,532.09; 18,410.20–20,844.04) and Botswana (15,687.15; 13,849.42–17,675.35). The age-standardized prevalence rate was the lowest in Albania (1.12; 0.68–1.81), followed by Syria Arab Republic (2.67; 1.91–4.09) and Bosnia and Herzegovina (3.02; 2.37–3.75). The age-standardized death rate was the highest in Lesotho (581.38; 500.13–710.70), followed by Eswatini (353.13; 307.81–417.71) and Mozambique (292.95; 244.05–377.11). Bosnia and Herzegovina had the lowest age-standardized death rate (0.04; 95% UI: 0.03–0.07), followed by Albania (0.06; 95% UI: 0.05–0.07) and Kuwait (0.06, 95% UI: 0.05–0.06). Lesotho (282,922.03; 24,609.45–35,669.26) had the highest age-standardized DALY rate, followed by Eswatini (18,325.88; 16,076.55–21,603.96) and Mozambique (15,888.85; 13,127.87–20,542.99). The age-standardized DALY rate in Bosnia and Herzegovina was the lowest at 2.15 (95% UI: 1.58–3.23), followed by Thailand (3.13; 95% UI: 2.70–3.69) and Egypt (3.52; 95% UI: 2.84–4.47) (Supplementary Table S1 and Figure 6).

From 1990 to 2019, the annual percentage change in age-standardized prevalence, death, and DALY rate differed, with Nepal (1,991.40; 260.37–24,730.34) having the largest increase, followed by Lao People's Democratic Republic (1,101.01; 251.38–23,424.18) and Mongolia 556.08 (0.00–0.00). The age-standardized death rates in Nepal (32,772.77; 1,818.56–538,830.21) had the highest increase, followed by Lao People's Democratic Republic (14,395.03; 726.13–107,208.08) and Madagascar (1,093.28; 453.79–6,618.79). Nepal (14,521.28; 858.14–174,846.49) had the largest increase in age-standardized DALYs, followed by Lao People's Democratic Republic (6,823.93; 853.20–77,769.47) and Madagascar (762.11; 345.57–3,308.26) (Supplementary Table S1).

### 3.4. Age

**Figure 7:**
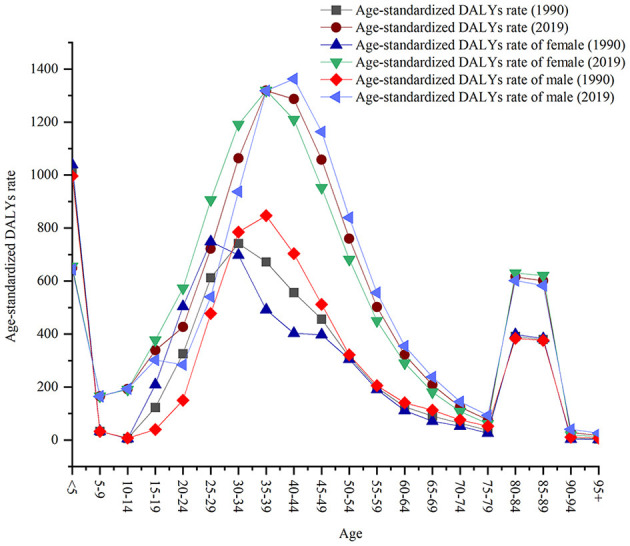
Global age-standardized DALY rates by age.

From 1990 to 2019, age-standardized DALY rates increased in all cases except for children under 5 years. In 2019, the global age-standardized DALY rate of HIV/AIDS began to increase after 5 years of age, reached a peak in the 35–39 age group, decreased with increasing age, and presented a small peak in the 80–89 age group (Figure 7).

The results showed that the age-standardized DALY rates of males and females showed a bimodal distribution, with a small peak in the 80–89 age and < 5 age groups in 1990 and 2019. In 2019, the age-standardized DALY rate was higher in women 10–35 years than in men and in men 40–89 years than in women. There were no significant differences in age-standardized DALY rates between men and women < 10 years of age and >89 years of age. In 1990, peaks were observed in females 25–29 years and in males 35–39 years. In 2019, the peaks were in the 35–39 age group for females and 40–44 age group for males (Figure 7).

**Figure 8:**
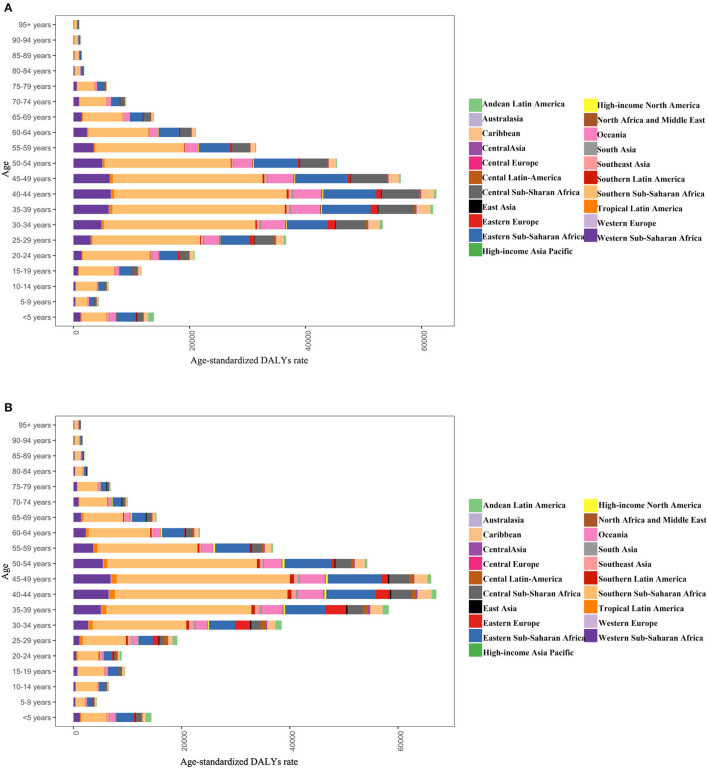
DALY age-standardized rates for males **(A)** and females **(B)** by age group for 21 disease burden regions in 2019.

**Figure 9:**
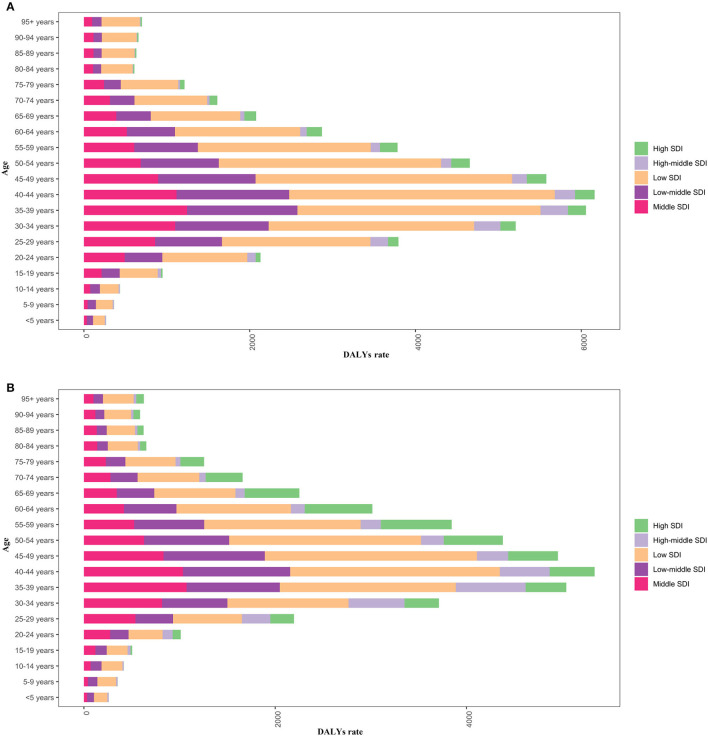
Male **(A)** and female **(B)** DALY rates by age in different SDI areas in 2019.

In 2019, there was little difference in disease burden between men and women across all regions and age groups, with both concentrated in sub-Saharan Africa in the 30–49 age group (Figure 8). Low SDI areas are represented in all age groups. In high and high-middle SDI areas, DALY rates were higher in males 25–79 years than in females (Figure 9).

### 3.5. Risk factors

**Figure 10:**
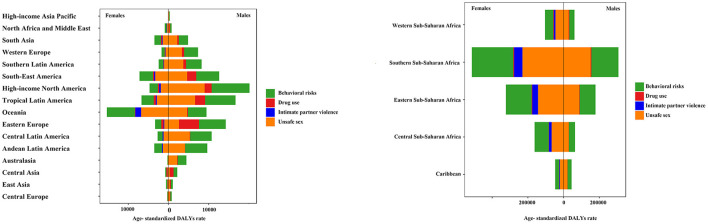
Risk factor analysis in 21 disease burden regions in 2019.

The major factors contributing to HIV/AIDS include behavioral risk, drug use, intimate partner violence, and unsafe sex, with different risk factors manifesting differently based on the region and age. Globally, unsafe sex remains dominant, with partner violence leading to higher DALY rates in women than in men, while drug use is more significant in men (Figure 10). In Africa, the main risk factors for HIV/AIDS originate from behavioral risk and unsafe sex (Figure 10).

**Figure 11:**
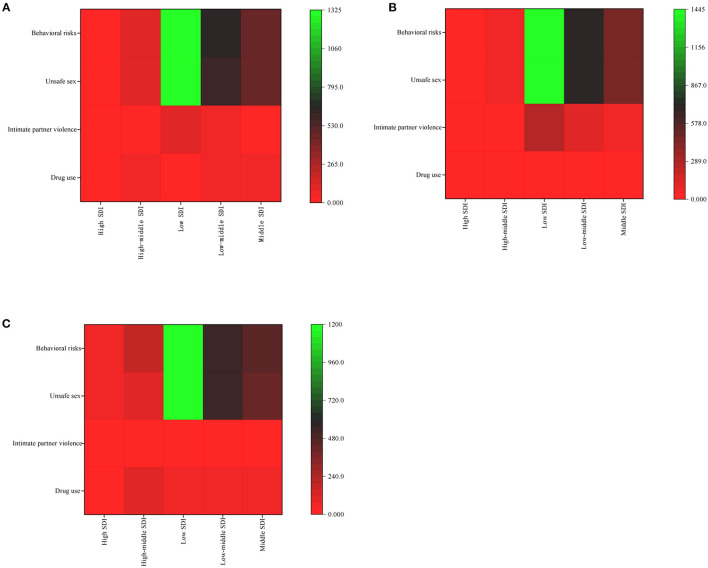
Analysis of risk factors for different SDI areas by both **(A)**, in males **(B)**, and females **(C)** in 2019.

HIV/AIDS infection was mainly attributed to behavioral risk and unsafe sex in low and medium SDI areas. In low SDI areas, women were more affected by partner violence than men, while men were less affected by substance use. The risk of HIV/AIDS infection due to drug use was higher among men in middle and high SDI areas than in other SDI areas (Figure 11).

## 4. Discussion

HIV/AIDS is a public health problem that requires considerable attention. In 1990–2019, the global HIV/AIDS prevalence, death, and DALY rates increased by 307.27 (95% UI: 304.45–312.63), 4.34 (95% UI: 3.77–4.89), and 221.91 (95% UI: 204.36–239.47), respectively, per 100,000 cases. Therefore, HIV/AIDS continues to impact human lives and health. Despite great efforts to scale up HIV/AIDS prevention and treatment worldwide, the trend of the HIV/AIDS epidemic is worsening.

Our findings showed that global HIV/AIDS DALY rates increased from 1990 to 2005 but decreased and leveled off after 2005 possibly due to (a) increased medical resources, (b) prevention and control measures (e.g., antiretroviral therapy), (c) elimination of mother-to-child transmission, which reduces the infection in children under 5 years, and (d) health education, which includes the promotion and use of condoms (15). The AIDS burden is mainly due to premature deaths. With treatment, HIV/AIDS patients live longer and have a normal life expectancy. Direct or indirect deaths in these patients may result from complications. Therefore, it is essential to adopt healthy lifestyle habits to reduce the burden of disease and control complications.

In infection rates remains high in females and occurs earlier than in males. The burden of prevalence, deaths, and DALYs caused by HIV/AIDS is considerable. Young women (15–24 years) in sub-Saharan Africa are particularly vulnerable. The mucosal area of women during sex is larger, resulting in a relatively high infection rate. During childbirth, the infant may be exposed to HIV/AIDS from the mother's bloodstream. Therefore, we must promote education and training, implement comprehensive prevention, and control policies, and strengthen interventions from the aspects of knowledge, behavior, and habits (16).

In areas with high and high-middle SDIs, the HIV/AIDS burden is higher in men than in women, which may be due to changes in people's sexual attitude and the increase in the number of men who have sex with men (MSM) and HIV/AIDS infection rates. Therefore, we should pay attention to the prevention of HIV/AIDS among MSM. The MSM population is listed as the key testing population to ensure that the funds are sufficient to carry out targeted research.

The burden of HIV/AIDS varies among different age groups. The burden of HIV/AIDS was higher in the 25–54 group, which may be due to the high prevalence of HIV/AIDS due to more active sexual life, MSM, commercial sex, and drug use. The prevalence of infections among infants and children under 5 years of age is high. The rates of mother-to-child transmission remain high despite effective prevention measures. Critical problems in HIV/AIDS prevention among infants and children are widespread. Several pregnant women living with HIV/AIDS cannot obtain adequate testing and treatment (17). In South Africa, the number of new infections among women of childbearing age (15–49 years old) was high in the past decade, and the increasing burden of HIV/AIDS infection among women had an additional impact on mother-to-child transmission (18). This creates an urgent need for effective plans to curb rising rates of HIV/AIDS. The global infection rates among infants and children under 5 years of age from 1990 to 2019 is high but declining. Much of this success depends on primary and secondary prevention, proper use of condoms, pre-exposure prophylaxis, and mother-to-child transmission. In addition, several regions have begun to pay attention to children. Sub-Saharan Africa, where the HIV/AIDS burden is high, has implemented novel treatment programs and policies for children (19). Increasing interventions for mothers and newborns, particularly for young women, women without formal education, women with poor socioeconomic status, and women in remote areas, strengthening HIV/AIDS media publicity, and improving the knowledge of HIV/AIDS can reduce the prevalence of HIV/AIDS to a certain extent (20). In the 80–89-year group, the DALY of HIV/AIDS shows another small peak, indicating that the aging of infected people is a common phenomenon in the society. Paying attention to the aging population has become a major public health challenge (21, 22). Previous studies showed that HIV/AIDS was the ninth leading cause of disease burden in the 0–9 group in 2019, and HIV/AIDS was the second leading cause of disease burden in the 25–49 group. The burden of HIV/AIDS increased significantly in the 10–24 group. It subsequently declined after the global scale-up of antiretroviral treatment. However, even though the HIV/AIDS burden has declined in recent years, it has yet to return to 1990 levels (23).

HIV/AIDS is prevalent in underdeveloped areas, and South Africa is still the major center of HIV/AIDS epidemic (24). The number of new infections has accounted for a high proportion in the world so far, and the high death rates have contributed to the economic burden in Africa. The emergence of this phenomenon is likely to be due to the lagging economic development in Africa and scarce medical resources. In Africa, it is a challenge to obtain equal healthcare (25), and education level is limited. There are multiple sexual partners and unprotected intercourse, exacerbating the risk of HIV infection (26). HIV/AIDS carriers accelerate the spread of the virus, resulting in the HIV/AIDS epidemic. Therefore, strengthening HIV/AIDS prevention and intervention in Africa can effectively reduce the burden of HIV/AIDS.

In areas with low SDIs, HIV/AIDS burden is high due to low economic development and limited access to effective healthcare and treatment. In areas with low SDIs, HIV/AIDS prevention is mainly dependent on state aid (27). International assistance is insufficient to meet the actual needs of some countries (28), and inadequate international support and domestic funding may lead to delays in treatment or the inability to seek treatment at all.

Previous studies have measured the global HIV/AIDS burden for DALYs 1900–2019; however, they did not investigate the risk factors that contribute to the burden of HIV/AIDS (3). The transmission of HIV is affected by multiple factors including social factors, which play an important role. Currently, in low SDI areas and some developing countries, especially in Africa, the effective response to HIV/AIDS transmission is not well developed, and the expected results in terms of prevention of mother-to-child transmission, community awareness, condom use, and drug use have not been achieved. Young women with HIV/AIDS are more vulnerable to sexual violence, rape, and transactional sex to obtain their daily needs, resulting in an increased risk of intimate partner violence (29). In men, drug and alcohol abuse and unsafe sexual practices are major causes. With this background, the media should actively promote HIV/AIDS awareness and identify the risk factors to increase overall awareness of HIV/AIDS.

HIV/AIDS prevention and treatment need to be addressed at the individual. The global response to HIV/AIDS requires the development and/or improvement of policies to ensure universal access to HIV/AIDS prevention, treatment, and support (30). Preventive strategies have been published elsewhere (31, 32). Such findings are essential to adjust the burden of life years and could provide the optimal prevention strategies and treatment options for people living with HIV/AIDS or at risk of infection (33).

## 5. Conclusion

This study analyzed the disease burden of HIV/AIDS in terms of temporal and spatial trends. Meanwhile, different measuring indexes of HIV/AIDS disease burden were analyzed. The HIV/AIDS burden varies based on the region, country, age, and sex. Trends in HIV/AIDS burden dramatically increased during 1990–2005, and then there was a decline. In countries with low SDI, HIV/AIDS age-standardized prevalence, death, and DALY rates remain high, especially in Africa, where the HIV/AIDS epidemic is worsening. This study indicated that the HIV/AIDS disease burden concentrated in the middle-aged population and that unsafe sex factors were dominant. This analysis could provide insights into the current trends of the HIV epidemic and develop suitable strategies to reduce the burden of HIV/AIDS.

## Data availability statement

The original contributions presented in the study are included in the article/Supplementary material, further inquiries can be directed to the corresponding author.

## Ethics statement

Ethical review and approval was not required for the study on human participants in accordance with the local legislation and institutional requirements. Written informed consent from the participants' legal guardian/next of kin was not required to participate in this study in accordance with the national legislation and the institutional requirements.

## Author contributions

NW contributed to the study design. JC and XW contributed to the data collection. YX, XZ, DH, HF, and WY contributed to the data analyses. XT and JC contributed to the interpretations and writing of the manuscript. All authors contributed to the manuscript proof. All authors read and approved the final manuscript.
