# Supplementary material for: Global, regional, and national HIV/AIDS disease burden levels and trends in 1990–2019: A systematic analysis for the global burden of disease 2019 study
Source: Front Public Health. 2023 Feb 15;11:1068664. doi: 10.3389/fpubh.2023.1068664 (PMC9975742; doi:10.3389/fpubh.2023.1068664)
Supplement: Supplementary file 1 [file Table_1.docx]

**Table S1** Prevalence cases, deaths, and disability-adjusted life years (DALYs) for HIV/AIDS in 2019 and percentage change in age standardised rates (ASRs) per 100000，in 204 countries，from 1990 to 2019.

|  | Prevalence（95%UI） | | |  | Deaths（95%UI） | | |  | DALYs（95%UI） | | |
| --- | --- | --- | --- | --- | --- | --- | --- | --- | --- | --- | --- |
| location | Number  （95% UI） | age-standardized  Rate  （95% UI） | precentage  Change in ASRs from 1990 to 2019 |  | Number  （95% UI） | age-standardized  Rate  （95% UI） | precentage  Change in ASRs from 1990 to 2019 |  | Number  （95% UI） | age-standardized  Rate  （95% UI） | precentage  Change in ASRs from 1990 to 2019 |
| Kazakhstan | 16742.87 (13231.76-21456.06） | 81.97 (64.94-104.87） | 5.18 (3.14-7.63) |  | 260.54 (246.37-276.00) | 1.28 (1.21-1.35） | 1.52 (1.35-1.71) |  | 15044.78 (14051.22-16143.44） | 29.66 (27.93-31.50） | 1.67 (1.49-1.89) |
| Latvia | 3794.85 (3140.66-4566.77） | 188.92 (150.70-236.83） | 7.54 (6.33-9.24) |  | 7.24 (7.10-7.38） | 5.55 (5.28-5.83） | 2.85 (2.59-3.12) |  | 5868 (5556.93-6236.88） | 463.13 (364.01-604.52) | 2.86 (2.61-3.13) |
| Niger | 38892.96 (28225.87-53101.26） | 282.9 (204.66-386.68） | 0.77 (-0.09-2.74) |  | 1423.11 1023.12-1962.63） | 10.83 (7.65-14.47） | 1.23 (0.07-3.48) |  | 76886.85 (55165.67-103723.26） | 517.77 (364.36-702.70) | 0.89 (-0.05-2.70) |
| Papua New Guinea | 89585.44 (2467.16-264840.32） | 1018.17 (28.05-2987.56） | 299.78 (26.53-603.51) |  | 66304.05 (53843.37-86579.82） | 45.23 (15.30-126.67） | 1034.38 (348.53-10291.57) |  | 203240.67 (53048.64-575723.27） | 2227.84 (637.39-6340.32) | 703.99 (275.26-1795.13) |
| Germany | 80178.59 (58182.93-105157.53） | 71.67 (49.31-98.89） | 0.43 (0.23-0.66) |  | 421.31 (402.16-442.48） | 0.39 (0.38-0.41） | -0.79 (-0.80- -0.78) |  | 23320.57 (20496.65-27979.50） | 214.5 (189.95-253.66) | -0.76 (-0.79- -0.72) |
| Colombia | 103026.65 (81298.88-130865.16） | 200.47 (157.58-256.40） | 7.10 (5.20-9.40) |  | 2449.69 (2334.69-2565.80） | 4.78 (4.56-5.01） | 1.76 (1.62-1.92) |  | 124897.84 (117873.87-132480.19） | 246.46 (232.54-261.43) | 1.86 (1.70-2.03) |
| United States of America | 1743127.93 (917751.32-2593838.54） | 432.45 (228.90-648.02） | 0.33 (0.07-0.56) |  | 7.36 (7.23-7.51） | 1.77 (1.75-1.78） | -0.82 (-0.82--0.82) |  | 415325.49 (346508.50-508431.60） | 108.87 (91.54-131.79） | -0.79 (-0.82--0.76) |
| Sri Lanka | 2023.43 (1713.4-2360.78） | 8.66 (7.3-10.07） | 1.91 （0.92-3.81） |  | 37.99 (26.7-58.32） | 0.17 (0.12-0.26) | -0.06 (-0.47-1.23) |  | 2220.55 (1575.12-3327.16） | 10.44 (7.18-16.13） | 0.03 (-0.41-1.29) |
| Samoa | 253.61 (13.9-1603.74） | 131.86 (7.17-865.29） | 3.45 （-0.48-27.2） |  | 34.03 (31.90-36.18) | 7.45 (0.03-59.35) | 6.34 (-0.79-126.37) |  | 704.59 (9.0-5571.78） | 371.4 (4.52-2959.53） | 5.66 (-0.68-83.17) |
| Ethiopia | 791550.25 (714482.82-881452.18） | 1031.62 (929.41-1135.86） | 0.12 （-0.33-1.02） |  | 4.02 (3.09-5.18) | 33.56 (28.68-39.58） | 0.07 (-0.39-0.91) |  | 1559170.28 (1245704.21-1962958.33） | 1699.06 (1423.54-2050.66） | -0.08 (-0.44-0.57) |
| North Macedonia | 113.95 (90.53-144.81） | 4.74 (3.70-6.21） | 7.23 （5.56-8.88） |  | 1.91 (1.60-2.19) | 0.10 (0.08-0.12） | 2.63 (1.93-3.57) |  | 123.69 (102.32-147.84） | 7.41 (5.89-9.23） | 3.40 (2.56-4.51) |
| Australia | 17305.78 (11205.10-23732.69） | 56.49 (35.5-79.28） | 0.12 （-0.06-0.31） |  | 70.65 (68.43-72.86) | 0.24 (0.23-0.25) | -0.88 (-0.89- -0.88) |  | 4293.99 (3653.3-5211.00） | 23.48 (20.84-28.03） | -0.86 (-0.88- -0.83) |
| Portugal | 39359.77 (32072.88-49547.42） | 320.74 (250.07-416.17） | 2.73 （2.05-3.93） |  | 570.3 (342.89-817.36） | 3.66 (3.48-3.86) | 0.81 (0.70-0.93) |  | 23606.2 (22057.68-25634.81） | 193.97 (180.94-210.97） | 0.73 (0.61-0.86) |
| Kyrgyzstan | 6191.71 (4364.42-9005.16） | 96.81 (69.75-137.79） | 10.59 （6.97-15.06） |  | 3.95 (0.02-31.88） | 2.65 (2.52-2.77） | 2.57 (2.33-2.81) |  | 9501.8 (8954.13-10162.12) | 144.93 (136.62-155.17） | 2.54 (2.29-2.83) |
| Northern Mariana Islands | 14.19 (6.73-26.99） | 33.1 (14.51-65.59） | 4.17 （1.51-9.43） |  | 31.78 (5.34-83.98） | 1.32 (0.21-3.25) | 5.33 (-0.07-20.68) |  | 29.61 (5.33-70.82) | 73.18 (12.43-182.51) | 4.88 (0.05-15.79) |
| Zambia | 1223914.46 (1129052.95-1331778.47） | 9223.97 (8523.07-9949.47） | 0.76 （0.25-1.65） |  | 22540.27 (19287.0-27224.58） | 164.92 (144.02-190.91） | -0.15 (-0.47-0.46） |  | 1361838.20 (1146734.17-1664684.03） | 8697.07 (7565.02-10233.22） | -0.20 (-0.48-0.25） |
| Turkmenistan | 3755.76 (2978.44-4963.33） | 70.02 (55.6-91.97） | 0.82 （0.48-1.34） |  | 1.71 (0.93-3.65） | 1.96 (1.84-2.09） | 0.00 (-0.07-0.08） |  | 5982.83 (5568.72-6451.76） | 110.66 (102.98-119.28） | 0.05 (-0.03-0.13） |
| Greece | 4407.02 (3334.74-6731.84） | 38.4 (28.79-56.64） | 2.11 （1.61-3.20） |  | 48.63 (46.44-50.99） | 0.2 (0.19-0.21） | -0.43 -0.47- -0.39） |  | 1378.82 (1228.38-1596.38） | 84.2 (63.93-109.02） | -0.32 (-0.39- -0.23） |
| Thailand | 527171.9 (463153.1-613722.19） | 623.33 (536.88-741.37） | 3.13 （2.19-5.16） |  | 37.11 (29.86-42.38） | 19.33 (14.6-29.79） | 9.39 （6.24-13.14） |  | 791963.81 (610836.87-1150689.80） | 951.85 (709.23-1498.01） | 6.02 (4.22-8.80） |
| Kenya | 1671885.95 (1484084.24-1878561.47） | 4235.34 (3733.04-4761.28） | 0.24 （0.06-0.50） |  | 51134.61 (45036.35-58977.47） | 133.48 (118.75-151.17） | 0.2 （-0.23-0.93） |  | 2736624.95 (2361566.77-3223934.30） | 6464.76 (5725.9-7385.10） | 0.02 （-0.29-0.47） |
| Grenada | 250.03 (209.85-299.75） | 226.37 (188.67-275.36） | 1.33 （0.62-1.93） |  | 5.46 (5.34-5.57） | 4.83 (4.73-4.93） | -0.52 （-0.53- -0.51） |  | 274.48 (263.40-288.52） | 248.68 (238.13-261.78） | -0.49 （-0.51- -0.46） |
| Lithuania | 1879.34 (1283.73-2380.19） | 63.26 (42.59-82.55） | 6.34 （0.00-9.87 |  | 5612.49 (4639.66-7197.49） | 1.88 (1.79-1.96） | 1.68 （1.51-1.86） |  | 2859.7 (2703.23-3042.21） | 200.43 (187.09-215.64） | 1.79 （1.61-2.00） |
| Barbados | 1507.43 (1330.42-1725.37） | 432.02 (371.05-519.98） | 1.51 （1.00-2.63） |  | 2.49 (2.37-2.61） | 7.1 (6.97-7.23） | -0.53 （-0.54--0.51） |  | 1205 (1159.42-1260.34） | 349.5 (19.74-2182.56） | -0.49 （-0.51--0.47） |
| Sierra Leone | 81465.21 (60138.45-110792.36） | 1199.64 (892.86-1627.06） | 8.87 （2.69-30.01） |  | 10237.54 (7790.54-13782.62） | 44.5 (35.58058.11） | 10.05 （3.55-22.91） |  | 152718.81 (118149.22-209311.88） | 2220.57 (1759.13-2943.39） | 8.68 （2.97-20.26） |
| Democratic People's Republic of Korea | 13630.92 (2166.63-79674.43） | 45.38 (7.23-265.81） | 8.47 （0.41-43.13） |  | 760.96 (15.88-4064.56） | 2.56 (0.06013.58） | 0.00  （0.00-0.00） |  | 37618.41 (1661.8101966-1.22） | 133.55 (5.61-678.84） | 15.13 （0.90-326.07） |
| Solomon Islands | 722.1 (34.95-4436.58） | 123.43 (5.89-795.96） | 2.82 （-0.56-25.96） |  | 40.57 (0.21-325.28） | 7.25 (0.04-59.74） | 5.14 （-0.81-95.18） |  | 2302.11 (25.28-17796.77） | 370.66 (4.22-2961.77） | 4.43 （-0.74-68.10） |
| Israel | 13364.74 (7299.85-19312.00） | 139.18 (77.21-200.06） | 5.11 （3.74-6.82） |  | 36.69 (35.15-38.29） | 0.38 (0.37-0.40） | -0.53 （-0.56--0.51） |  | 2687.59 (2164.94-3342.60） | 28.58 (23.10-35.65） | -0.33 （-0.44--0.18） |
| Nigeria | 1963043.74 (1758881.02-2206517.82） | 1269.29 (1135.99-1417.91） | 3.37 （2.16-5.09） |  | 20.62 (0.51-157.22） | 54.11 (44.15-66.62） | 4.29 （2.67-6.05） |  | 4393501.59 (3409027.24-5629468.37） | 2618.12 (2105.56-3276.18） | 3.63 （2.32-5.10） |
| Uganda | 1366481.42 (1212028.13-1518281.65） | 5029.57 (4542.77-5551.89） | -0.4 （-0.49--0.29） |  | 20762.27 (16892.62-26840.11） | 72.63 (60.48-90.53） | -0.88 （-0.90--0.83） |  | 1280470.09 (1033764.80-1652760.04） | 3925.56 (3242.21-4909.40） | -0.87 （-0.90-0.83） |
| Jamaica | 15823.58 (12693.99-19650.11） | 524.65 (422.94-650.27） | 2.84 （1.81-4.32） |  | 100.43 (98.66-102.17） | 14.08 (13.84-14.34） | 0.5 （0.46-0.54） |  | 20870.04 (20231.88-21676.95） | 706.25 (684.95-732.13） | 0.49 （0.45-0.54） |
| Honduras | 9252.56 (7947.30-10976.85） | 109.51 (91.08-133.13） | 1.30 （0.66-2.21） |  | 91.39 (68.24-121.53） | 1.01 (0.77-1.31） | -0.53 （-0.65- -0.28） |  | 5797.33 （4361.06-7813.19） | 61.55 (47.06-80.88） | -0.47 （-0.59--0.29） |
| Brunei Darussalam | 542.54 (288.61-881.87） | 105.59 (57.41-169.95） | 6.74 （5.05-8.74） |  | 55.97 (53.46-58.53） | 1.14 (1.00-1.30） | 2.18 （1.31-3.56） |  | 302.6 （250.29-370.51） | 62.55 (51.97-76.08） | 2.57 （1.62-3.91） |
| Taiwan (Province of China) | 5136.79 (2473.09-8887.18） | 18.14 (8.63-31.69） | 6.78 （3.70-9.43） |  | 5602.85 （4492.25-7429.45） | 0.57 (0.52-0.62） | 13.11 （11.57-14.82） |  | 8098.84 （7380.05-8890.43） | 29.35 (26.71-32.21） | 11.21 （9.88-12.82） |
| Chad | 134079.93 (103761.69-171922.98） | 1288.2 (1013.31-1633.63） | 1.86 （0.32-5.60） |  | 4695.32 （3225.40-6822.24） | 46.21 (32.63-64.76） | 2.16 （0.51-4.99） |  | 264864.74 （182538.82-385370.91） | 2276.38 (1580.15-3258.27） | 1.73 （0.31-4.14） |
| Nepal | 27605.46 (2238.160105538.74） | 96.29 (8.19-371.56） | 1911.4 （260.37-24730.34） |  | 1433.8 （24.41-8641.66） | 5.13 (0.09-31.22） | 32772.77 （1818.56-538830.21） |  | 75499.32 （1751.59-440500.01） | 1212.63 (1004.7401504.31） | 14521.28 （858.14-174846.49） |
| Bermuda | 180.05 (157.88-198.78） | 259.38 (225.74-308.96） | 0.25 （-0.07-0.63） |  | 6.25 （6.13-6.38） | 7.53 (7.38-7.69） | -0.65 （-0.66- -0.64） |  | 274.97 （265.67-284.53） | 372.59 (359.79-388.04） | -0.63 （-0.65--0.62） |
| Belize | 2657.73 (2149.67-3328.93） | 651.02 (548.23-789.99） | 2.44 （1.82-3.16） |  | 418.00 （410.60-425.41） | 20.18 (19.79-20.61） | -0.02 （-0.04-0.01） |  | 4309.62 （4159.55-4503.35） | 1052.94 (1017.63-1098.95） | 0.01 （-0.02-0.05） |
| San Marino | 23.36 (5.39-68.68） | 61.65 (12.18-188.13） | 0.42 （-0.51-3.36） |  | 0.67 （0.16-2.08） | 1.54 (0.33-4.91） | 0.29 （-0.58-2.96） |  | 27.88 （5.82-90.72） | 70.86 (12.73-235.69） | 0.11 （-0.62-2.71） |
| Belgium | 23813.75 (12953.80-36400.98） | 162.27 （89.97-243.02） | 4.78 （3.32-7.45） |  | 5.45 （5.22-5.70） | 0.41 (0.39-0.42） | -0.65 （-0.67- -0.63） |  | 4076.4 （3132.35-5279.18） | 30.92 (24.68-38.72） | -0.49 （-0.59--0.36） |
| Fiji | 472.09 (396.61-561.85） | 50.86 (42.56-60.78） | 4.50 （2.72-7.97） |  | 8.81 （5.99-13.75） | 0.95 (0.64-1.48） | 1.97 （0.65-5.96） |  | 560.42 （366.25-860.70） | 13.01 (11.68-14.79） | 2.08 （0.79-5.15） |
| Uzbekistan | 22344.82 (13096.14-31621.82） | 62.79 (36.61-87.04） | 7.59 （3.50-14.40） |  | 584.56 （553.98-617.10） | 1.67 (1.58-1.76） | 1.03 （0.90-1.18） |  | 31682.02 （29720.02-33943.13） | 88.89 (83.39-95.02） | 1.05 （0.90-1.21） |
| Italy | 96494.94 (67739.98-131230.26） | 124.08 (83.72-173.67） | 0.49 （0.17-0.92） |  | 620.89 （606.95-634.56） | 0.77 (0.75-0.78） | -0.76 （-0.77- -0.75） |  | 32627.04 （29168.44-37797.77） | 43.52 (38.92-50.20） | -0.77 （-0.79- -0.74） |
| Brazil | 891346.98 (765069.10-1044866.74） | 366.46 (313.83-430.32） | 2.27 （1.37-3.28） |  | 15560.83 （15297.00-15837.29） | 6.39 (6.28-6.50） | 0.13 （0.11-0.16） |  | 807432.7 （773530.58-849402.57） | 337.12 (323.13-354.69） | 0.04 （0.00-0.08） |
| Lebanon | 1202.09 (136.77-4143.43） | 22.46 (2.55-76.66） | 0.06 （-0.76-3.53） |  | 61.11 （1.52-283.03） | 1.15 (0.03-5.34） | -0.27 （-0.95-2.69） |  | 3567.65 （92.39-15921.75） | 67.22 (1.70-310.93） | -0.20 （-0.94-2.80） |
| Bulgaria | 1775.78 (1575.20-1983.07） | 23.96 (20.21-27.70） | 4.36 （0.00-7.56） |  | 162.86 （152.58-172.85） | 0.57 (0.54-0.60） | 0.86 （0.74-1.01） |  | 2170.07 （2037.23-2311.55） | 289.36 (273.20-308.69） | 0.87 （0.74-1.02） |
| South Africa | 7921791.53 （7450923.15-8394891.07） | 13577.41 （12784.2-14340.97） | 36.51 （30.05-43.91） |  | 1002.61 （586.88-1647.47） | 250.91 (219.70-302.51） | 31.05 （22.28-43.76） |  | 7682976.67 （6492018.56-9535635.39） | 13060.16 (11237.23-15907.93） | 23.47 18.3 29.85） |
| Maldives | 16.34 (12.44-23.58） | 3.12 (2.47-4.18） | 0.72 （0.47-1.04） |  | 1.65 （1.47-1.90） | 0.17 (0.15-0.18） | 2.50 （1.88-3.17） |  | 47.34 （42.57-53.36） | 8.51 (7.69-9.40） | 2.04 （1.57-2.57） |
| Argentina | 188657.25 (110526.54-283951.37） | 395.33 (229.43-598.12） | 2.67 （1.94-3.49） |  | 2047.56 （1681.88-2546.64） | 3.68 (3.60-3.76） | 1.11 （1.05-1.17） |  | 102487.76 （90831.09-121183.19） | 363.67 (350.83-377.41） | 1.17 （0.98-1.48） |
| Slovakia | 345.79 (261.72-456.78） | 5.4 (4.05-7.24） | 9.73 (0.00-21.35） |  | 4.14 （3.44-4.94） | 0.07 (0.06-0.08） | 0.00  (0.00-0.00) |  | 230.01 （183.91-278.01） | 4.34 (3.25-5.41） | 0.00  (0.00-0.00) |
| Congo | 96895.21 (66388.72-143898.43） | 2004.76 （1380.65-2977.20） | -0.01 (-0.42-0.85） |  | 4274.98 （3509.99-5284.39） | 91.42 （76.9-111.24） | -0.43 （-0.62- -0.14) |  | 234944.1 （191121.85-292627.72） | 4649.59 （3857.99-5689.52） | -0.47 （-0.63--0.24） |
| Saint Vincent and the Grenadines | 437.27 (371.36-534.62） | 367.52 （305.23-458.05） | 0.81 (0.43-1.43） |  | 19.96 （19.54-20.35） | 16.17 （15.83-16.48） | -0.10 （-0.13- -0.08） |  | 985.08 （953.70-1022.92） | 3.52 （2.84-4.47） | -0.06 （-0.09- -0.03） |
| New Zealand | 2561.76 (1579.06-3524.63） | 53.37 （31.90-74.78） | 1.37 (0.65-1.87） |  | 1850.07 （1763.03-1946.28） | 0.18 （0.18-0.19） | -0.87 （-0.88- -0.87) |  | 599.67 （498.64-732.63） | 13.07 （10.9-16.08） | -0.81 （-0.84--0.77） |
| United Kingdom | 131957.31 (74519.36-189985.03） | 168.23 （94.76-243.37） | 4.51 (3.74-5.51） |  | 7052.74 （6970.28-7132.64） | 0.3 (0.30--0.31） | -0.60 (-0.60- -0.59) |  | 20297.23 (15281.27-27101.55) | 27.34 （21.00-36.17） | -0.34 （-0.48--0.17） |
| American Samoa | 13.96 (6.71-26.91） | 26.69 （12.76-52.43） | 3.25 （1.21-7.48） |  | 0.48 （0.09-1.29） | 0.93 （0.17-2.51) | 3.57 （-0.34-18.57) |  | 26.19 （5.35-69.21) | 50.32 （10.11-134.06) | 3.57 （-0.22-14.71) |
| Burkina Faso | 93881.34 (79977.96-111594.56） | 585.2 （503.97-696.56） | -0.82 （-0.85- -0.76） |  | 13637.73 （11588.87-16582.82） | 18.11 （14.46-22.82) | -0.9 （-0.93- -0.83) |  | 171529.39 （137901.70-219319.30) | 3251.35 （1368.31-6625.54) | -0.90 （-0.93- -0.85) |
| Niue | 2.48 （0.17-9.75) | 148.68 （9.81-591.63) | 20.61 （2.14-68.80) |  | 683.18 （276.88-1810.87) | 8.58 （0.13-41.13) | 28.44 （0.64-143.83) |  | 7.14 （0.15-34.15) | 428.12 （9.47-2081.96) | 25.54 （0.84-119.06) |
| Togo | 109713.66 (89445.94-133932.98) | 1697.38 （1385.91-2055.05) | 3.67 （0.93-12.70) |  | 27125.17 （22630.13-33798.61) | 54.75 （41.79-71.44) | 3.82 （1.22-8.87) |  | 181954.7 （137531.46-243662.54) | 2655.81 （2011.38-3514.00) | 3.01 （0.84-7.28) |
| Dominican Republic | 59038.17 (45273.89-76699.40) | 541.14 （417.72-697.25) | 1.34 （0.34-3.30) |  | 1362.08 （982.51-2015.46) | 12.65 （9.17-18.46) | 1.45 （0.23-3.96) |  | 67468.33 （47468.95-101764.40) | 614.24 （435.32-920.26) | 0.99 （0.05-2.87) |
| Namibia | 205367.31 (192574.27-218770.87) | 10105.38 （9494.90-10787.74) | 10.85 （8.09-14.65) |  | 3949.35 （43.61-22919.25) | 187.83 （162.72-221.60) | 7.69 （4.63-12.17) |  | 203600.16 （176728.53-240231.71) | 9404.07 （8253.30-10986.48) | 6.00 （3.85-8.84) |
| Rwanda | 207718.9 (185780.29-229835.97) | 2169.44 （1955.04-2394.77) | 0.98 （-0.19-5.23) |  | 231.43 （226.99-236.06) | 32.72 （27.71-38.85) | 0.00 （-0.63-1.33) |  | 164310.2 （139269.12-202865.52) | 1602.7 （1377.23-1926.41) | -0.14 （-0.66-0.92) |
| Norway | 3454.35 (2264.21-5034.09) | 60.88 （39.29-89.23) | 3.75 （2.78-4.84) |  | 13.69 （13.41-13.99) | 0.23 （0.22-0.23) | -0.68 （-0.69- -0.67) |  | 908.61 （773.68-1096.42) | 16.22 （13.81-19.53) | -0.55 （-0.61- -0.46) |
| Armenia | 1389.42 (1111.43-1824.16) | 39.74 （31.61-53.04) | 32.07 （0.00-37.47) |  | 19.08 （18.11-20.13) | 0.56 （0.53-0.59) | 15.33 （14.21-16.49) |  | 1062.97 （994.76-1146.73) | 31.44 （29.47-33.86) | 13.63 （12.10-17.89) |
| Costa Rica | 6763.89 (5540.15-8458.71) | 129.7 （105.72-163.45) | 2.23 （1.65-3.30) |  | 145.61 （138.67-152.31) | 2.82 （2.69-2.95) | 0.30  （0.22-0.37) |  | 7504.21 （7102.70-7954.55) | 147.89 （140.23-156.61) | 0.29 （0.21-0.37) |
| Japan | 45514.43 (24022.55-70332.23) | 24.56 （13.74-36.83） | 7.56 （5.33-9.88） |  | 160.69 （157.64-163.60） | 0.09 （0.09-0.09） | 0.93 （0.88-0.97） |  | 9061.09 （7116.04-11543.93） | 5.98 （4.96-7.27） | 1.28 （0.99-1.60） |
| Madagascar | 42078.57 (20597.6-72319.51) | 186.79 （92.33-325.37） | 244.01 （104.37-781.33） |  | 117.88 （112.22-123.50） | 12.03 （7.93-17.36） | 1093.28 （453.79-6618.79） |  | 138152.75 （86732.97-205950.54） | 600.41 （386.58-876.69） | 762.11 （345.57-3308.26） |
| Oman | 2695.04 (1946.62-3515.21) | 51.48 （38.32-67.57） | 8.76 （5.17-14.08） |  | 317.68 （5.31-1503.16） | 2.24 （1.22-3.58） | 7.48 （3.83-12.32） |  | 4964.3 （2899.38-7335.44） | 98.93 （57.88-151.15） | 6.69 （3.51-10.66） |
| Croatia | 900.83 (710.11-1102.54) | 16.57 （13.12-21.23） | 4.86 （2.97-6.55） |  | 0.15 （0.00-0.73） | 0.15 （0.14-0.15） | 0.21 （0.14-0.30） |  | 386.67 （353.25-426.71） | 8.33 （7.74-9.09） | 0.31 （0.21-0.43） |
| Egypt | 4438.19 (3717.86-5258.31) | 4.76 （3.95-5.54） | 1.17 （0.73-1.66） |  | 55.92 （45.19-72.38） | 0.06 （0.05-0.08） | -0.48 （-0.61--0.11） |  | 3517.97 2845.00-4508.73） | 18.73 （15.02-24.54） | -0.43 （-0.56- -0.15） |
| Bolivia (Plurinational State of) | 16491.88 (3194.72-57065.20) | 140.27 （28.11-486.21） | 0.77 （-0.70-7.96） |  | 561.15 （337.88-804.43） | 6.10 （0.35-38.28） | 0.57 （-0.90-15.59） |  | 43983.1 （2317.31-277947.65） | 591.18 （313.26-852.91） | 0.73 （-0.88-14.48） |
| C么te d'Ivoire | 425741.44 (377183.21（477303.78) | 2071.43 （1843.92-2319.03) | 0.00  （0.00-0.00） |  | 160.33 （2.91-928.84) | 67.64 （56.70-80.01) | 0.00  （0.00-0.00） |  | 751134.27 （624383.25-938531.20） | 3309.02 （2810.28-3982.69） | 0.00  （0.00-0.00） |
| Timor-Leste | 3632.73 (176.57（20621.85) | 311.33 （17.18-1799.89) | 1.61 （-0.50-27.32） |  | 138.19 （128.53-148.87） | 21.15 （0.20-131.00） | 9.95 （-0.34-88.75） |  | 13515.33 （140.70-81098.20） | 1086.16 （12.92-6354.92） | 7.04 （-0.36-69.79） |
| Saint Kitts and Nevis | 508.41 (153.97-1145.10） | 740.82 （225.50-1655.87） | 5.92 （0.16-17.98） |  | 4772.85 （3813.23-6048.05） | 44.59 （7.55-116.05） | 10 （0.35-35.82） |  | 1466.83 （259.69-3643.86） | 217.43 （4.48-1525.42） | 8.58 （0.14-29.20） |
| Benin | 70465.1 (56692.91-84859.74） | 795.49 （656.96-951.86） | 12.43 （8.18-19.29） |  | 2033.47 （1494.34-2748.98） | 22.78 （17.15-29.76） | 23.12 （13.00-43.22） |  | 116818.82 （86677.68-158770.11） | 905.55 （734.43-1141.52） | 16.04 （9.80-27.12） |
| Mexico | 219624.5 (176922.56-272317.39） | 167.37 （135.39-206.36） | 2.61 （2.12-3.41） |  | 5034.32 （4976.08-5091.25） | 3.85 （3.80-3.89） | 0.31 （0.29-0.33） |  | 264700.56 （256166.76-276125.71） | 201.82 （195.29-210.50） | 0.34 （0.31-0.39） |
| Nicaragua | 14404.45 (11377.18-17441.43） | 222.4 （178.56-268.44） | 25.12 （15.95-35.34） |  | 27.35 （20.32-36.35） | 8.82 （5.15-12.88） | 14.61 8.18-22.19） |  | 31035.68 （20076.77-42075.50） | 468.91 （296.47-646.09） | 13.36 （7.77-19.70） |
| Guinea-Bissau | 31191.26 (17877.49-47984.86） | 2139.69 （1264.96-3261.74） | 6.99 （3.85-12.06） |  | 54.57 （27.62-123.01） | 73.58 （41.72-121.54） | 10.56 （5.52-19.95） |  | 55062.07 （32146.69-91019.71） | 3637.35 （2136.52-5971.48） | 8.26 （4.44-14.89） |
| Montenegro | 87.16 (70.60-107.73） | 12.75 （9.90-16.64） | 5.25 （3.43-6.94 ） |  | 47.86 （29.10-103.37） | 0.23 （0.20-0.28） | 0.77 （0.44-1.20） |  | 80.68 （68.62-96.02） | 15.53 （12.71-18.63） | 1.09 （0.67-1.63） |
| Kiribati | 39.67 (28.39-54.06） | 34.06 （25.62-45.19） | 0.65 （0.28-1.12） |  | 540.43 （515.95-566.01） | 1.29 （0.98-1.72） | 0.61 （0.02-1.41） |  | 108.65 （81.30-139.44） | 366.82 （3.15-2903.00） | 0.61 （.10-1.21） |
| Tunisia | 3031.64 (578.94-11265.60） | 24.48 （4.69-91.29） | 8.84 （2.03-30.09） |  | 147.79 （6.62-811.61） | 1.16 （0.06-6.33） | 7.96 （-0.12-40.09） |  | 7506.35 （451.43-40592.70） | 60.73 （3.77-329.59） | 7.54 （0.03-34.86） |
| Guinea | 123874.73 (89847.69-168216.90） | 930.02 （675.98-1314.81） | 7.58 （4.20-13.27） |  | 4037.04 （2810.43-5857.31） | 45.81 （32.64-64.22） | 8.84 （4.88-15.19） |  | 222774.68 （153189.40-322986.06） | 2290.98 （1612.15-3266.80） | 7.42 （4.16-12.60） |
| Afghanistan | 5125.30 (455.35-19470.24） | 1350.17 （991.98-1805.70） | 2.29 （-0.59-21.55） |  | 14.46 （11.36-19.42） | 0.96 （0.02-4.90） | 1.63 （-0.93-25.24） |  | 20686.24 （363.16-98247.49） | 53.15 （1.06-251.49） | 1.80 （-0.92-22.48） |
| United States Virgin Islands | 235.73 (208.82-264.74） | 16.63 （1.56-64.16） | 0.86 （0.32-1.79） |  | 7.86 （7.70-8.00） | 6.46 （6.33-6.58） | -0.08 （-0.11- -0.05） |  | 347.75 （336.92-359.27） | 315.18 （305.17-326.83） | -0.07 （-0.1- -0.03） |
| Malaysia | 56215.47 (47718.02-65428.83） | 215.89 （190.25-247.28） | 5.42 （3.78-6.90） |  | 3829.61 （1169.65-10720.02） | 4.90 （3.13-6.82） | 17.98 （8.08-33.40） |  | 93229.36 （58933.28-128695.85） | 73.88 （69.06-79.31） | 15.08 （8.03-24.75） |
| Malawi | 1006346.08 (935370.58-1078897.49） | 165.65 （140.59-191.45） | 0.64 （0.17-1.50） |  | 2958.91 （2388.71-3703.64） | 111.20  （97.02-131.68） | -0.37 （-0.61-0.10） |  | 844095.41 （711157.14-1041010.20） | 5835.24 （5060.20-6934.59） | -0.41 （-0.61- -0.05） |
| Saint Lucia | 364.83 (313.07-453.18） | 7818.28 （7299.41-8379.99） | 1.31 （0.76-1.77） |  | 1.60 （1.21-2.10） | 3.53 （3.46-3.59） | -0.53 （-0.54- -0.52） |  | 370.25 （354.80-390.24） | 822.93 （796.78-855.81） | -0.49 （-0.51- -0.46） |
| Burundi | 85495.43 （75575.65 98394.06） | 183.17 （153.92-235.09） | -0.71 （-0.88-0.38） |  | 0.12 （0.00-0.57） | 23.90 （19.11-31.41） | -0.82 （-0.94--0.55） |  | 120838.74 （99735.97-149726.63） | 18.72 （3.75-108.02） | -0.84 （-0.94- -0.59） |
| Canada | 92282.82 （61219.05-127156.15） | 1030.5 （900.84-1183.58） | 0.71 （0.36-1.18） |  | 253.56 （242.05-266.13） | 0.58 （0.55-0.61） | -0.77 （-0.78--0.76） |  | 17763.28 （14703.76-22254.64） | 42.03 （34.99-52.72） | -0.69 （-0.74- -0.63） |
| Slovenia | 274.48 （155.20-458.57） | 203.08 （130.82-291.30） | 5.91 （0.00-10.72） |  | 2529.73 （1645.60-3727.56） | 0.09 （0.09-0.10） | -0.53 （-0.56- -0.49） |  | 122 （108.89-140.17） | 5.74 （5.20-6.47） | -0.49 （-0.54- -0.42） |
| Democratic Republic of the Congo | 379897.3 （307560.62-471196.06） | 10.55 （5.99-17.90） | -0.51 （-0.64--0.31） |  | 1.84 （0.36-5.02） | 15.46 （11.76-20.97） | -0.74 （-0.83--0.55） |  | 593942.49 （450748.68-802319.03） | 796.38 （611.33-1060.22） | -0.75 （-0.83- -0.59） |
| Paraguay | 15841.16 （13013.73-19375.59） | 576.06 （470.16-707.67） | 11.30 （6.65-16.10） |  | 7.51 （7.14-7.91） | 8.47 （5.06-12.23） | 8.30 （3.99-19.29） |  | 32742.22 （20865.10-44099.59） | 477.07 （300.62-650.60） | 7.90 （4.06-16.62） |
| Libya | 1471.75 （224.21-7039.10） | 228.87 （185.01-275.62） | 2.55 （-0.37-12.17） |  | 98.45 （55.48-151.31） | 1.15 （0.02-6.41） | 4.46 （-0.82-31.79） |  | 4570.36 （164.15-25311.72） | 62.09 （2.12-350.83） | 3.91 （-0.74-24.00） |
| Seychelles | 105.74 （86.72-137.63） | 19.37 （2.94-89.38） | 3.34 （2.82-4.14） |  | 60.74 （58.08-63.62） | 3.33 （2.55-4.27） | 6.39 （2.54-12.32） |  | 192.89 （149.23-253.66） | 1087.26 （801.81-1488.01） | 5.54 （2.46-9.50） |
| Cyprus | 192.56 （113.20-302.53） | 90.82 （73.66-119.43） | 2.98 （2.14-3.92） |  | 4.63 （4.22-5.08） | 0.27 （0.25-0.30） | 2.69 （1.50-4.27） |  | 205.53 （179.08-239.88） | 12.86 （11.26-14.96） | 2.62 （1.46-4.08） |
| Iran (Islamic Republic of) | 27608.06 （21290.42-34582.99） | 12.44 （7.19-19.67） | 6.51 （4.43-8.87） |  | 1176.65 （851.73-1714.50） | 1.26 （0.91-1.82） | 13.02 （6.55-41.77） |  | 64050.29 （47949.43-88444.97） | 69.04 （51.03-94.60） | 11.82 （6.53-27.61） |
| Qatar | 149.98 （125.91-183.54） | 28.54 （21.84-35.22） | -0.26 （-0.45-0.06） |  | 4.29 （3.59-5.03） | 0.14 （0.12-0.16） | -0.66 （-0.73--0.59） |  | 234.77 （196.48-273.21） | 82.84 （30.17-257.38） | -0.69 （-0.74--0.61） |
| Pakistan | 74378.19 （6584.61-359119.89） | 4.67 （4.16-5.24） | 3.71 （-0.54-27.48） |  | 2.16 （2.03-2.30） | 2.02 （0.02-12.39） | 5.11 （-0.90-140.63） |  | 234938.81 （4924.63-1384164.51） | 109.69 （2.36-637.74） | 5.3 （-0.82-69.43） |
| Comoros | 118.6 （67.86-395.47） | 37.05 （3.24-182.90） | 2.08 （0.80-9.43） |  | 26590.89 （21751.56-33029.40） | 0.28 （0.00-2.09） | 17.01 （-0.31-2520.52） |  | 129.75 （25.10-782.13） | 13.03 （12.22-13.92） | 6.03 （0.62-47.49） |
| Turkey | 5281.18 （3881.45-7520.94） | 17.74 （10.16-57.95） | 9.54 （0.00-9.55） |  | 3065.06 （2606.60-3735.32） | 0.26 （0.21-0.31） | 0.00  （0.00-0.00） |  | 11780.18 （9541.81-14090.16） | 14.38 （11.46-17.34） | 0.00  （0.00-0.00） |
| Philippines | 287664.05 （147105.34-500581.50） | 5.81 （4.26-8.29） | 0.00 （0.00-0.00） |  | 5222.48 （5136.05-5314.86） | 4.62 （4.54-4.70） | 2.5 （2.43-2.58） |  | 337510.38 （316549.05-378449.21） | 290.35 （271.65-327.62） | 3.08 （2.80-3.58） |
| Tajikistan | 4528.95 （3441.58-5548.36） | 252.48 （131.32-439.24） | 5.65 （2.76-9.67） |  | 60.02 （45.02-87.39） | 0.67 （0.51-0.98） | 2.57 （1.21-6.13） |  | 3425.98 （2602.55-4834.31） | 37.05 （28.18-52.50） | 2.61 （1.35-5.31） |
| Bosnia and Herzegovina | 132.81 （92.47-172.96） | 51.49 （38.48-63.24） | 1.37 （0.73-1.93） |  | 18.86 （17.85-19.88） | 0.04 （0.03-0.07） | -0.46 （-0.64- -0.06） |  | 79.65 （52.96-138.97） | 2.15 （1.58-3.23） | -0.39 （-0.54- -0.13） |
| Hungary | 2069 （1654.04-2404.22） | 3.02 （2.37-3.75） | -0.28 （-0.52- -0.04） |  | 37.25 （34.89-39.73） | 0.28 （0.27-0.30） | -0.78 （-0.8--0.77） |  | 1620.42 （1514.03-1730.64） | 163.65 （128.72-212.71） | -0.78 （-0.8- -0.76） |
| Serbia | 2302.65 （1805.78-3192.20） | 15.53 （12.73-17.82） | 2.66 （0.00-2.25） |  | 5.29 （4.63-6.01） | 0.51 （0.32-1.06） | 0.00  （0.00-0.00） |  | 2431.14 （1552.10-5037.69） | 28.42 （18.59-56.15） | 0.00  （0.00-0.00） |
| Sweden | 5099.08 （2682.36-7597.20） | 23.11 （17.56-33.56） | 1.21 （0.68-1.67） |  | 20.41 （19.83-21.03） | 0.17 （0.17-0.18） | -0.76 （-0.77- -0.74） |  | 1272.93 （1063.08-1561.27） | 29.4 （22.94-37.94） | -0.68 （-0.72- -0.61） |
| Switzerland | 22508.06 （14420.08-30746.17） | 44.78 （23.64-67.33） | 0.71 （0.32-1.06） |  | 36.89 （35.02-38.67） | 0.34 （0.32-0.35） | 7.31 （6.78-7.86） |  | 3251.60 （2467.39-4268.20） | 7.30 （6.03-8.50） | 1.41 （0.87-2.21） |
| Haiti | 166904.29 （135935.61-206367.54） | 185.46 （118.31-255.11） | 0.16 （-0.35-1.69） |  | 5908.15 （4655.48-7676.80） | 54.64 （44.05-69.60） | 0.10 （-0.40-0.90） |  | 310940.58 （238572.66-411152.47） | 2671.5 （2103.49-3449.95） | -0.06 （-0.46-0.64） |
| Uruguay | 11896.96 （8723.05-15638.47） | 1475.86 （1210.82-1812.76） | 4.46 （2.87-7.14） |  | 177.38 （173.61-181.36） | 4.51 （4.41-4.62） | 2.25 （2.15-2.35） |  | 8401.76 （7857.30-9179.76） | 228.51 （213.20-250.36） | 2.56 （2.40-2.76） |
| Trinidad and Tobago | 9388.78 （8351.14-10908.44） | 330.96 （231.63-443.96） | 2.27 （1.33-4.12） |  | 206.42 （202.23-210.30） | 12.9 （12.64-13.14） | 0.44 （0.40-0.48） |  | 10655.63 （10292.96-11117.77） | 371.68 （357.78-388.47） | 0.48 （0.42-0.53） |
| Yemen | 6578.16 （706.85-25548.67） | 592.51 （520.91-708.12） | 1.03 （-0.69-10.10） |  | 1115.21 （786.16-1600.26） | 1.24 （0.03-6.65） | 0.85 （-0.94-26.05） |  | 21887.81 （577.72-111770.86） | 69.27 （2.08-347.73） | 0.92 （-0.92-18.55） |
| Bhutan | 724.42 （47.87-3401.19） | 24.27 （2.62-93.23） | 2.56 （-0.63-40.45） |  | 30.65 （0.54-207.13） | 4.00 （0.07-25.68） | 3.04 （-0.88-91.13） |  | 1717.12 （34.25-11791.22） | 181.17 （158.88-211.12） | 3.15 （-0.85-71.17） |
| India | 1826474.48 （1632416.53-2051258.53） | 88.61 （6.24-412.76） | 17.13 （9.08-29.99） |  | 346.70 （6.55-1776.31） | 3.26 （2.89-3.72） | 28.72 （17.29-43.49） |  | 2593725.92 （2258833.25-3056817.19） | 260.96 （6.03-1511.83） | 18.36 （12.35-24.99） |
| Lesotho | 374039.93 （351294.84-399651.16） | 127.95 （114.43-143.25） | 12.44 （8.35-18.70） |  | 10792.65 （9013.96-13646.02） | 581.38 （500.13-710.70） | 16.93 （9.63-27.03） |  | 571734.82 （472939.99-724172.24） | 28922.04 （24609.45-35669.26） | 13.23 （8.25-19.89） |
| Cabo Verde | 2807.05 （1557.59-4911.21） | 19532.09 （18410.20-20844.04） | 0.66 （-0.23-3.48） |  | 1078.27 （708.18-1576.95） | 10.11 （5.17-22.36） | -0.02 （-0.62-2.16） |  | 2944.99 （1495.58-6465.44） | 526.55 （272.12-1159.63） | -0.09 （-0.64-1.82） |
| Zimbabwe | 1227095.76 （1148849.07-1311033.63） | 504.86 （289.85-862.11） | 1.67 （0.17-9.42） |  | 3.10 （0.21-14.26） | 180.62 （163.86-200.22） | 0.33 （-0.46-2.16） |  | 1174444.8 （1052610.33-1337151.63） | 9176.82 （8369.90-10219.61） | 0.15 （-0.49-1.71） |
| Ghana | 306961.88 （259942.21-364559.89） | 10490.93 （9862.95-11204.35） | 1.14 （0.55-2.14） |  | 14620.93 （11988.57-18358.14） | 55.64 （46.93-67.75） | 1.66 （0.77-2.97） |  | 763384.77 （612085.02-976303.41） | 2701.11 （2238.54-3356.92） | 1.29 （0.59-2.30） |
| China | 551426.02 （286453.52-1027215.40） | 1115.68 （947.34-1329.92） | 4.62 （2.35-7.30） |  | 237.58 （2.33-1420.30） | 1.74 （1.43-2.05） | 6.24 （3.28-27.56） |  | 1396028.83 （1120500.34-1700876.29） | 82.28 （66.65-98.84） | 5.23 （2.95-18.58） |
| Micronesia (Federated States of) | 898.55 （25.42-6516.64） | 31.72 （16.48-59.31） | 19.81 （0.62-151.36） |  | 45.36 （1.13-228.69） | 49.69 （1.18-257.10） | 9.98 （-0.64-49.60） |  | 2230.17 （95.74-11519.49） | 2359.74 （100.35-11824.04） | 5.89 （-0.63-27.89） |
| Estonia | 3212.75 （2781.86-3659.83） | 949.69 （26.83-6909.46） | 146.39 （113.52-177.04） |  | 24.41 （23.31-25.54） | 2.56 （2.40-2.72） | 70.84 （65.38-76.82） |  | 2041.76 （1894.45-2189.42） | 8.35 （7.69-9.13） | 67.63 （61.59-75.18） |
| Vanuatu | 346.39 （24.17-2170.66） | 241.08 （206.51-277.54） | 3.12 （-0.41-24.52） |  | 45.91 （43.26-48.94） | 7.77 （0.04-62.50） | 5.82 （-0.78-112.29） |  | 1031.95 （15.06-8153.64） | 377.6 （5.42-3002.79） | 4.75 （-0.67-71.35） |
| Russian Federation | 1137793.59 （938202.14-1401106.67） | 130.19 （8.98-855.05） | 23.43 （17.02-30.75） |  | 7.36 （5.70-10.23） | 11.39 （11.27-11.50） | 4.29 （4.22-4.36） |  | 1071665.71 （1030498.81-1124364.35） | 672.64 （647.09-707.04） | 4.43 （4.28-4.63） |
| Ukraine | 264448.2 （203327.88-335088.72） | 700.01 （571.01-877.21） | 9.25 （5.94-12.27） |  | 1.35 （1.18-1.59） | 12.63 （12.08-13.20） | 3.69 （3.44-3.96） |  | 346668.22 （327331.93-367497.41） | 675.61 （637.63-716.14） | 3.78 （3.51-4.05） |
| Georgia | 4510.62 （3074.20-6794.12） | 515.98 （383.87-664.21） | 60.86 （42.27-86.86） |  | 23.28 （22.19-24.50） | 0.58 （0.56-0.61） | 16.19 （15.08-17.37） |  | 1503.9 （1330.88-1823.15） | 19.14 （15.63-21.66） | 16.00 （14.19-19.27） |
| Singapore | 4650.52 （3049.78-6865.71） | 104.47 （68.61-167.83） | 2.39 （1.73-3.63） |  | 35.99 33.46-38.77） | 0.45 （0.42-0.48） | 0.80 （0.63-0.98） |  | 1896.51 （1639.28-2382.70） | 15.16 （13.04-18.34） | 0.98 （0.74-1.29） |
| France | 102792.77 （68949.31-141153.58） | 58.88 （38.24-87.62） | 0.07 （-0.12-0.25） |  | 456.98 （434.35-480.32） | 0.57 （0.55-0.60） | -0.88 （-0.89- -0.88） |  | 26242.59 （22693.03-30919.14） | 34.51 （30.12-40.51） | -0.87 （-0.88- -0.85） |
| Ireland | 2224.39 （1389.21-3283.12） | 122.73 （78.22-172.31） | 3.26 （2.05-5.07） |  | 8.63 （8.19-9.08） | 0.16 （0.16-0.17） | -0.51 （-0.54- -0.47） |  | 607.2 （520.07-729.87） | 11.84 （10.19-14.13） | -0.36 （-0.45--0.25） |
| Austria | 17405.05 （9761.48-25652.72） | 41.5 （25.23-61.61） | 3.52 （0.00-5.23） |  | 35.47 （33.73-37.25） | 0.34 （0.32-0.35） | -0.59 （-0.62- -0.56） |  | 2883.76 （2221.85-3801.93） | 28.32 （22.34-36.33） | -0.37 （-0.50--0.21） |
| Netherlands | 21047.54 （14264.25-27074.39） | 158.1 （89.74-231.08） | 0.59 （0.41-0.78） |  | 678.89 （640.20-717.56） | 0.24 （0.23-0.25） | -0.87 （-0.88- -0.86） |  | 3783.72 （2980.17-4766.55） | 18.83 （15.17-23.49） | -0.8 （-0.84--0.76） |
| Myanmar | 215613.5 （194444.22-238029.34） | 94.22 （63.37-123.39） | 18.45 （17.29-19.59） |  | 31746.08 （25775.17-37448.33） | 8.38 （6.66-10.67） | 86.83 （48.78-158.57） |  | 264399.32 （208433.47-344842.06） | 93.76 （88.37-99.83） | 48.5 （36.94-63.64） |
| Mongolia | 418.59 （163.84-836.54） | 381.27 （343.16-421.14） | 556.08 （0.00-0.00） |  | 15.03 （1.78-38.02） | 0.46 （0.05-1.18） | 0.00  （0.00-0.00） |  | 689.74 （104.87-1720.00） | 19.93 （2.92-49.69） | 0.00  （0.00-0.00） |
| Viet Nam | 247483.93 （202240.19-326645.78） | 12.68 （4.88-25.00） | 6.85 （5.76-8.44） |  | 6399.44 （4916.18-8629.51） | 5.60 （4.31-7.50） | 12.78 （7.44-26.08） |  | 365851.93 （286869.20-480542.44） | 322.12 （253.15-422.83） | 9.39 （6.26-15.60） |
| Czechia | 1671.27 （1224.86-2210.93） | 220.36 （180.00-292.82） | 11.23 （8.28-14.31） |  | 6643.09 （6353.09-6948.76） | 0.15 （0.14-0.15） | 2.27 （2.04-2.48） |  | 944.41 （871.15-1031.14） | 60.12 （39.13-92.60） | 2.30 （2.06-2.59） |
| Marshall Islands | 71.57 （2.36-454.72） | 12.94 （9.25-18.34 | 3.17 （-0.56-36.87） |  | 105.37 （99.05-112.62） | 7.31 （0.04-59.33） | 5.68 （-0.80-107.41） |  | 210.16 （1.84-1653.29） | 61.73 （56.30-67.73） | 5.07 （-0.75-84.23） |
| Albania | 31.98 （20.06-50.58） | 126.86 （4.19-830.68） | 0.45 （0.02-0.97） |  | 2117.65 （1318.14-3471.89） | 0.06 （0.05-0.07） | 0.38 （0.12-0.85） |  | 82.55 （72.08-97.22） | 3.13 （2.70-3.69） | 0.44 （0.19-0.85） |
| Poland | 15674.11 （9560.67-27520.79） | 1.12 （0.68-1.81） | 15.73 （10.32-23.14） |  | 134.09 （131.53-136.75） | 0.30 （0.30-0.31） | 4.93 （4.79-5.08） |  | 7631.01 （7016.76-8749.69） | 18.20 （16.83-20.74） | 4.97 （4.65-5.53） |
| Romania | 14583.45 （12823.62-16688.09） | 34.18 （20.65-60.85） | 16.23 （11.86-23.91） |  | 142.17 （135.79-149.26） | 0.77 （0.73-0.81） | -0.26 （-0.31--0.21） |  | 8502.6 （7945.24-9127.61） | 50.20 （46.78-54.02） | -0.40 （-0.45--0.34） |
| Belarus | 17047.4 （12752.63-22347.05） | 78.07 （67.53-91.61） | 17.21 （0.00-29.64） |  | 293.71 （276.68-311.86） | 2.69 （2.54-2.86） | 2.01 （1.80-2.22） |  | 15966.29 （14889.80-17207.45） | 24.17 （20.97-30.11） | 2.27 （2.02-2.53） |
| Iceland | 193.4 （158.98-255.26） | 158.73 （116.12-212.69） | 1.64 （1.11-3.14） |  | 0.88 （0.84-0.93） | 0.24 （0.23-0.25） | -0.68 （-0.70- -0.65） |  | 57.43 （51.17-65.45） | 16 （14.29-18.14） | -0.59 （-0.64--0.54） |
| Luxembourg | 557.89 （345.46-845.20） | 50.83 （41.17-68.12） | 1.66 （1.16-2.32） |  | 0.86 （0.78-0.95） | 0.34 （0.32-0.35） | -0.72 （-0.74--0.70） |  | 160.27 （137.33-193.55） | 22.23 （19.27-26.55） | -0.65 （-0.69--0.59） |
| Malta | 405.48 （247.46-580.98） | 72.33 （43.91-110.16） | 4.33 （3.25-5.36） |  | 1.41 （1.33-1.48） | 0.28 （0.27-0.30） | -0.55 （-0.58- -0.51） |  | 100.13 （81.96-126.79） | 21.26 （17.63-26.73） | -0.34 （-0.45- -0.19） |
| Guyana | 9394.47 （7997.78-11018.03） | 80.75 （47.93-117.99） | 4.82 （2.67-7.69） |  | 180.82 （177.53-184.07） | 23.51 （23.08-23.93） | 0.72 （0.68-0.77） |  | 9837.98 （9473.74-10259.37） | 1266.15 （1218.88-1320.51） | 0.89 （0.81-0.96） |
| Antigua and Barbuda | 279.15 （239.00-316.72） | 1232.09 （1051.24-1437.96） | 1.06 （0.41-1.75） |  | 80.03 （78.43-81.76） | 7.09 （6.97-7.23） | -0.45 （-0.46- -0.43） |  | 366.08 （353.10-379.47） | 1328.96 （1283.96-1389.41） | -0.4 （-0.42- -0.38） |
| Bahamas | 4114.2 （3570.03-4951.17） | 279.35 （240.53-320.52） | 0.65 （0.30-1.35） |  | 116.15 （113.85-118.48） | 26.56 （26.05-27.06） | -0.01 （-0.03-0.02） |  | 5718.92 （5522.29-5979.15） | 691.12 （667.55-720.67） | -0.02 （-0.06-0.01） |
| Peru | 83192.53 （65458.4-113567.04） | 951.81 （817.28-1160.65） | 3.84 （2.43-4.99） |  | 3447.66 （2580.1-4561.94） | 7.03 （4.08-11.48） | 1.75 （0.51-3.82） |  | 149951.47 （88195.72-252681.37） | 442.05 （258.58-751.16） | 2.06 （0.75-4.41） |
| Suriname | 3542.16 （3045.90-4273.62） | 236.71 （187.75-319.20） | 2.68 （1.64-3.87） |  | 732.08 （40.53-4632.63） | 16.55 （16.26-16.85） | -0.07 （-0.09- -0.05） |  | 5173.60 （5011.20-5386.90） | 869.87 （842.60-907.10） | -0.03 （-0.06-0.00） |
| Ecuador | 39422.75 （29255.28-54698.16） | 588.8 （499.06-724.56） | 7.28 （5.88-9.10） |  | 1207.31 （1128.86-1291.29） | 6.91 （6.46-7.39） | 7.93 （7.22-8.75） |  | 65720.82 （60947.97-71319.07） | 369.93 （343.79-401.72） | 7.2 （6.51-8.02） |
| Morocco | 13334.1 （1475.76-43095.84） | 221.06 （167.12-301.23） | 2.70 （-0.19-10.55） |  | 494.28 （12.97-3033.72） | 1.36 （0.03-8.25） | 2.18 （-0.78-17.84） |  | 28770.65 （1130.38-164296.81） | 79.73 （2.97-464.65） | 2.05 （-0.73-14.60） |
| Saudi Arabia | 10595.04 （4358.22-24950.72） | 35.65 （3.92-115.46） | 1.47 （0.15-4.98） |  | 14226.62 （12204.20-17576.51） | 1.68 （0.65-4.84） | 2.24 （0.38-7.47） |  | 35556.81 （13999.80-96901.19） | 6.52 （5.10-8.39） | 2.02 （0.33-6.41） |
| Eritrea | 25050.18 （18334.41-34114.98） | 23.98 （9.88-59.74） | -0.14 （-0.66-2.02） |  | 5546.61 （4439.29-7431.36） | 27.04 （19.76-36.03） | 0.45 （-0.37-2.32） |  | 73886.43 （50267.35-105283.74） | 1301.64 （938.12-1771.64） | 0.23 （-0.46-1.94） |
| Greenland | 190.39 （97.23-306.89） | 472.09 （353.15-632.43） | 0.75 （0.30-1.23） |  | 1.62 （1.29-1.97） | 2.52 （2.02-3.00） | -0.37 （-0.58- -0.06） |  | 88.48 （68.63-110.41） | 149.58 （117.40-186.10） | -0.24 （-0.48-0.05） |
| Central African Republic | 114176.74 （88127.37-150654.53） | 295.39 （149.96-479.05） | -0.06 （-0.39-0.58） |  | 1415.55 （940.70-2006.02） | 141.84 （115.86-185.49） | 0.85 （0.14-1.79） |  | 290237.99 （225468.73-396495.04） | 6810.68 （5458.63-9043.27） | 0.47 （-0.04-1.16） |
| Mauritania | 1201.03 （518.59-4948.71） | 2764.82 （2148.87-3583.35） | -0.71 （-0.89-0.19） |  | 23172.25 （19920.18-28094.46） | 0.69 （0.02-5.23） | -0.79 （-0.98-3.65） |  | 1283.4 （206.79-8091.84） | 40.00 （6.76-253.52） | -0.76 （-0.97-0.96） |
| Puerto Rico | 8073.09 （6432.74-9165.37） | 40.27 （17.37-161.19） | -0.43 （-0.59- -0.26） |  | 2401.05 （1393.51-3905.46） | 5.45 （5.35-5.57） | -0.79 （-0.8- -0.79） |  | 10268.39 （9899.86-10604.62） | 261.55 （252.25-271.16） | -0.81 （-0.82- -0.81） |
| Botswana | 350982.81 （307488.48-400883.26） | 209.96 （172.20-248.86） | 3.20 （2.17-4.65） |  | 143850.63 （123860.99-177382.31） | 258.09 （216.64-320.45） | 1.39 （0.50-2.80） |  | 290130.91 （236339.25-373733.50） | 12487.43 （10432.61-15741.47） | 0.95 （0.30-1.87） |
| Cameroon | 587763.36 （535496.61-642506.58） | 15687.15 （13849.42-17675.35） | 5.1 （3.11-8.32） |  | 13.46 （0.07-107.02） | 109.67 （96.96-127.76） | 7.34 （4.16-11.80） |  | 1230774.38 （1048184.57-1507579.41） | 5271.58 （4624.36-6257.43） | 5.90 （3.48-9.13） |
| Sao Tome and Principe | 27.80 （21.97-42.43） | 2560.94 （2318.52-2809.59） | -0.63 （-0.71- -0.38） |  | 263.55 （17.60-1943.65） | 0.16 （0.09-0.27） | -0.52 （-0.78-0.29） |  | 23.99 （15.31-36.19） | 13.31 （8.78-19.52） | -0.56 （-0.73- -0.29） |
| Eswatini | 213547.41 （197725.90-231546.31） | 16.71 （13.19-26.35） | 69.12 （34.42-112.26） |  | 3321.36 （2892.32-3991.05） | 353.13 （307.81-417.71） | 110.95 （39.87-259.35） |  | 187679.46 （162078.5-227533.95） | 18325.88 （16076.55-21603.97） | 65.36 （28.90- -125.65） |
| Gambia | 25410.14 （16636.78-36472.08） | 22031.68 （20432.73-23878.50） | 11.86 （5.78-23.14） |  | 1602.89 （1242.65-2132.73） | 67.57 （45.48-97.17） | 22.47 （11.16-41.69） |  | 57444.02 （37299.25-84669.90） | 3286.17 （2227.12-4724.27） | 18.01 （9.19-32.90） |
| Liberia | 37782.43 （27232.39-53080.03） | 1470.19 （981.57-2088.82） | 3.88 （0.69-23.26） |  | 1651.95 （1277.25-2203.36） | 43.18 （33.20-56.39） | 4.73 （1.33-11.60） |  | 85891.41 （65478.34-116351.14） | 1141.54 （863.24-1495.88） | 3.94 （1.07-10.35） |
| Mali | 110557.99 （81758.48-146836.68） | 743.43 （558.47-1001.32） | 2.28 （0.16-9.96） |  | 4495.76 （3328.85-5870.16） | 31.45 （23.48-40.62） | 3.21 （0.57-8.62） |  | 246636.26 （183252.02-329591.55） | 305.07 （184.91-426.33） | 2.67 （0.38-7.31） |
| Palau | 29.56 （1.99-120.72） | 141.87 （9.04-577.96） | 20.87 （2.19-69.53） |  | 1.86 （0.03-8.98） | 8.22 （0.12-38.90） | 27.43 （0.70-142.09） |  | 87.87 （1.80-423.44） | 411.48 （8.50-1989.40） | 24.8 （0.96-113.48） |
| Tokelau | 1.99 （0.14-7.94） | 153.11 （10.56-618.15） | 19.64 （2.01-64.82） |  | 8.7 （8.47-8.94） | 8.92 （0.14-43.21） | 29.43 （0.63-150.42） |  | 5.91 （0.13-28.21） | 447.23 （10.06-2163.87） | 25.99 （0.95-115.91） |
| Spain | 103330.41 （87643.79-121126.98） | 169.59 （142.46-200.72） | 0.12 （-0.04-0.36） |  | 232.79 （231.26-234.28） | 1.09 （1.03-1.15） | -0.8 （-0.81- -0.79） |  | 36241.97 （32872.33-39834.50） | 11.67 （9.82-14.25） | -0.81 （-0.82- -0.79） |
| Cuba | 24596.43 （15479.89-35982.32） | 181.29 （111.27-268.48） | 19.00 （14.11-27.12） |  | 337.47 （329.71-345.73） | 2.57 （2.51-2.64） | 3.71 （3.57-3.84） |  | 17444.11 （16272.13-19124.20） | 141.22 （132.09-153.88） | 3.87 （3.59-4.25） |
| Chile | 55504.71 （32762.70-85378.20） | 271.94 （158.17-424.80） | 4.75 （3.47-6.20） |  | 492.01 （480.54-503.89） | 2.35 （2.29-2.40） | 1.86 （1.77-1.96） |  | 27181.87 （24563.93-32191.03） | 133.45 （120.63-158.29） | 1.88 （1.64-2.28） |
| Indonesia | 290263.09 （252122.16-339060.58） | 104.27 （90.42-121.95） | 124.05 （0.00-144.70） |  | 17214.73 （13128.19-25526.44） | 2.11 （1.67-2.81） | 0.00  （0.00-0.00） |  | 419399.7 （339282.24-523824.05） | 298.97 （18.88-2032.78） | 0.00  （0.00-0.00） |
| Guatemala | 29371.29 （19712.50-46967.74） | 184.73 （130.80-283.72） | 2.37 （1.57-3.67） |  | 668.77 （638.27-701.12） | 4.16 （3.99-4.36） | -0.03 （-0.09-0.03） |  | 38571.64 （36150.52042023.66） | 225.08 （211.27-245.27） | 0.06 （-0.01-0.15） |
| Dominica | 212.11 （182.96-249.09） | 295.95 （252.76-353.47） | 1.56 （0.91-2.27） |  | 5.01 （4.91-5.12） | 6.89 （6.74-7.03） | -0.28 （-0.30--0.26） |  | 260.31 （250.530271.34） | 373.24 （359.52-389.39） | -0.22 （-0.25- -0.19） |
| Cambodia | 70832.73 （50386.56-90691.28） | 463.3 （336.86-581.89） | 30.1 （17.27-58.41） |  | 3772.47 （3273.18-4434.59） | 9.07 （5.99-12.93） | 115.65 （40.13-353.33） |  | 75139.96 （49347.14-111805.88） | 466.99 （310.95-675.46） | 65.58 （30.66-144.53） |
| Republic of Moldova | 8289.32 （5813.00-11576.34） | 184.11 （127.39-261.47） | 10.53 （6.14-18.81） |  | 0.29 （0.15-0.48） | 3.66 （3.43-3.88） | 2.17 （1.93-2.45） |  | 8797.91 （8205.49-9468.64） | 160.38 （126.69-203.15） | 2.27 （2.01-2.57） |
| Tonga | 41.79 （17.86-88.55） | 45.71 （19.60-97.25） | 3.72 （1.31-9.13） |  | 3188.21 （2332.20-4335.00） | 2.04 （0.38-5.54） | 5.88 （0.12-25.95） |  | 96.82 （20.98-267.26） | 103.21 （22.23-280.57） | 5.1 （0.28-18.38） |
| Gabon | 49558.56 （38133.70-65185.69） | 3142 （2454.92-4087.02） | 5.14 （0.88-20.00） |  | 1.86 （0.02-13.85） | 72.44 （52.05-103.04） | 3.11 （0.48-8.36） |  | 58414.98 （41501.24-84492.33） | 3580.88 （2583.34-5128.86） | 2.57 （0.30-7.58） |
| Mozambique | 2355345.93 （2031689.97-2778668.78） | 10533.23 （9209.70-12263.13） | 15.94 （8.98-27.79） |  | 18681.54 （18480.09-18876.36） | 292.95 （244.05-377.11） | 15.21 （8.53-25.54） |  | 4102654.72 （3278719.32-5395265.04） | 15888.85 （13127.87-20542.99） | 12.9 （7.27-21.75） |
| Senegal | 47356.4 （38429.67-59331.63） | 410.18 （334.17-508.44） | 2.08 （0.89-4.31） |  | 7.01 （2.96-15.01） | 14.22 （10.87-18.77） | 2.27 （0.87-4.42） |  | 85347.43 （65488.53-115016.19） | 695.95 （538.16-925.75） | 1.81 （0.66-3.61） |
| Guam | 116.19 （52.40-237.85） | 69.44 （30.70-145.24） | 1.49 （0.29-3.94） |  | 0.85 （0.21-2.58） | 4.07 （1.71-8.68） | 3.25 （0.29-16.32） |  | 358.17 （144.26-761.42） | 213.52 （84.63-450.75） | 3.27 （0.33-14.64） |
| Kuwait | 297.04 （246.15-351.78） | 5.77 （4.91-6.64） | 0.82 （0.53-1.19） |  | 2.87 （2.52-3.27） | 0.06 （0.05-0.06） | -0.58 （-0.64- -0.51） |  | 183.04 （161.67-208.14） | 3.76 （3.30-4.27） | -0.47 （-0.55- -0.39） |
| Tuvalu | 17.68 （1.27-71.72） | 152.23 （10.83-628.81） | 19.61 （2.13-64.24） |  | 0.98 （0.02-4.76） | 8.66 （0.13-42.03） | 29.18 （0.64-149.63） |  | 49.81 （1.21-243.23） | 436.53 （10.24-2126.55） | 25.56 （0.98-116.00） |
| El Salvador | 20679.28 （16954.81-24551.90） | 339.65 （279.56-401.00） | 7.31 （3.65-12.35） |  | 680.25 （362.18-975.67） | 11.36 （5.97-16.39） | 4.56 （1.91-7.12） |  | 35701.28 （19145.16-50935.94） | 186.14 （178.41-196.30） | 4.57 （1.80-7.01） |
| Algeria | 9485.27 （1533.30-33141.14） | 22.07 （3.61-75.47） | 6.06 （1.16-21.37） |  | 2798.23 （2199.78-3757.80） | 0.62 （0.04-4.59） | 2.01 （-0.63-13.25） |  | 13482.13 （1170.08-91815.80） | 30.64 （2.66-209.41） | 1.62 （-0.64-10.41） |
| Bahrain | 243.66 （206.95-299.83） | 12.64 （10.75-15.39） | -0.05 （-0.23-0.21） |  | 482.07 （457.19-508.78） | 0.38 （0.30-0.51） | -0.5 （-0.65- -0.21） |  | 362.23 （281.00-499.34） | 440.76 （8.57-2120.65） | -0.51 （-0.64- -0.27） |
| Cook Islands | 28.26 （1.92-110.99） | 157.4 （10.21-635.44） | 23.23 （2.55-80.60） |  | 1.71 （0.02-8.12） | 8.88 （0.13-42.96） | 29.31 （0.80-154.47） |  | 79.34 （1.55-371.49） | 2130.65 （383.71-5367.92） | 26.93 （0.88-128.65） |
| Mauritius | 3400.37 （2903.32-4211.69） | 230.4 （193.13-291.80） | 38.52 （33.40-45.62） |  | 91.38 （85.14-98.01） | 6.05 （5.64-6.50） | 4.97 （4.42-5.59） |  | 4509.88 （4210.33-4876.82） | 310.35 （288.65-336.07） | 5.33 （4.76-5.98） |
| Sudan | 106199.62 （32755.69-292417.00） | 303.74 （94.37-807.24） | 7.03 （0.22-80.53） |  | 5154.88 （2590.53-10324.19） | 16.08 （8.30-31.48） | 13.3 （2.25-80.19） |  | 271457.18 （131399.49-558494.99） | 786.51 （387.66-1592.26） | 11.27 （1.82-69.55） |
| South Sudan | 112694.11 （41715.2-239723.33） | 1619.93 （602.91-3484.01） | 8.32 （1.22-46.39） |  | 4390.88 （1896.72-8709.59） | 65.00 （27.83-132.83） | 11.40 （2.28-40.10） |  | 236846.6 （101045.05-472958.40） | 38.36 （34.07-47.04） | 9.74 （1.85-36.12） |
| Lao People's Democratic Republic | 8575.81 （3068.55-36825.71） | 124.96 （44.79-521.02） | 1101.01 （251.38-23424.18） |  | 373.86 （2.90-2708.03） | 5.65 （0.04-41.22） | 14395.03 （726.13-107208.08） |  | 21218.89 （1314.11-141979.50） | 150.51 （140.48-162.32） | 6823.93 （853.20-77769.47） |
| Andorra | 109.29 （6.96-453.02） | 107.37 （6.45-439.16） | 0.45 （-0.73-6.14） |  | 82269.51 （64383.37-103942.82） | 2.59 （0.18-11.61） | 0.18 （-0.76-4.32） |  | 129.97 （8.75-574.33） | 119.25 （7.60-509.60） | 0.07 （-0.78-4.23） |
| Venezuela (Bolivarian Republic of) | 92235.14 （72734.72-110791.61） | 306.01 （238.57-371.35） | 6.83 （5.59-8.83） |  | 1378.25 （982.51-1892.77） | 6.08 （5.80-6.39） | 0.60 （0.52-0.70） |  | 94923.85 （89339.4-101110.31） | 316.41 （298.22-337.21） | 0.54 （0.45-0.63） |
| Nauru | 15.21 （1.05-61.94） | 151.87 （10.50-625.14） | 20.60 （2.26-69.17） |  | 0.82 （0.01-3.99） | 8.82 （0.13-41.96） | 29.65 （0.79-149.84） |  | 44.57 （1.06-222.54） | 443.49 （10.44-2145.36） | 26.6 （1.17-119.51） |
| Republic of Korea | 29976.86 （17559.23-47045.64） | 40.71 （24.30-61.75） | 6.26 （0.00-7.85） |  | 1776.98 （1737.57-1815.01） | 0.18 （0.17-0.19） | 0.42 （0.30-0.55） |  | 8886.09 （6634.78-12553.85） | 12.56 （9.66-17.03） | 0.92 （0.49-1.71） |
| Panama | 20996.73 （16764.16-27477.35） | 497.33 （397.08-649.65） | 3.51 （2.77-4.76） |  | 24.76 （24.29-25.22） | 12.81 （12.22-13.42） | 0.68 （0.57-0.82） |  | 28975.46 （27361.72-30826.77） | 687.40 （648.90-732.34） | 0.80 （0.67-0.94） |
| Iraq | 2449.81 （1178.77-5161.48） | 5.76 （2.91-12.41） | 8.31 （4.18-18.10） |  | 82.18 （16.47-317.55） | 0.19 （0.04-0.76） | 4.00 （0.15-13.92） |  | 5450.07 （1047.94-21765.25） | 12.28 （2.36-48.66） | 4.30 （0.36-13.16） |
| Monaco | 27.61 （6.88-82.48） | 64.13 （12.74-199.49） | 0.55 （-0.48-3.86） |  | 0.59 （0.09-1.40） | 1.58 （0.33-5.12） | 0.29 （-0.57-2.92） |  | 33.32 （7.80-107.23） | 73.02 （13.53-241.97） | 0.07 （-0.62-2.62） |
| United Arab Emirates | 2299.28 （207.77-10041.26） | 46.69 （3.14-256.26） | 15.71 （0.58-103.77） |  | 19.03 （0.10-156.14） | 4.32 （0.03-29.87） | 25.97 （-0.67-219.24） |  | 7282.41 （190.42-40371.44） | 114.14 （1.99-673.92） | 12.32 （-0.59-98.95） |
| Angola | 383909.75 （256109.12-531017.18） | 1699.88 （1162.27-2347.38） | 19.35 （7.92-53.37） |  | 1520.83 （997.26-2096.22） | 77.73 （55.01-110.16） | 28.86 （13.29-59.51） |  | 923172.14 （624640.61-1355275.55） | 3880.77 （2701.61-5566.83） | 24.66 （11.27-52.20） |
| Somalia | 39195.12 （23368.10-63465.69） | 253.07 （140.91-425.14） | 9.18 （2.18-30.68） |  | 226.22 （181.62-264.93） | 22.01 （16.35-29.96） | 62.7 （16.96-559.49） |  | 182032.05 （131550.89-251850.72） | 3355.68 （2834.72-4096.83） | 41.96 （11.82-204.75） |
| Denmark | 6519.08 （4570.91-8912.36） | 91.37 （61.66-127.01） | 1.08 （0.79-1.40） |  | 16801.83 （11489.49-24344.12） | 0.34 （0.33-0.36） | 7.5 （7.01-8.03） |  | 1547.95 （1268.57-1932.91） | 23.21 （19.27-28.84） | 2.49 （1.86-3.37） |
| United Republic of Tanzania | 1503310.3 （1366722.03-1660045.81） | 3607.68 （3275.54-3954.77） | -0.11 （-0.27-0.14） |  | 171.08 （156.95-186.27） | 65.29 （55.07-78.53） | -0.61 （-0.73- -0.40） |  | 1540235.55 （1276764.91-1929095.31） | 2072.9 （1596.51-2763.27） | -0.64 （-0.74- -0.48） |
| Jordan | 611.7 （497.90-812.96） | 5.73 （4.76-7.14） | 5.72 （3.08-8.90） |  | 84.89 （1.43-471.03） | 0.25 （0.18-0.34） | 3.32 （1.97-5.00） |  | 1602.46 （1228.00-2133.77） | 13.88 （10.52-18.43） | 2.79 （1.68-4.12） |
| Palestine | 190.82 （108.59-339.08） | 3.72 （2.15-6.60） | 2.66 （1.71-3.96） |  | 13.62 （11.63-15.99） | 0.29 （0.25-0.34） | 5.22 （3.75-7.65） |  | 830.37 （684.44-1003.12） | 16.67 （13.88-20.26） | 5.11 （3.71-7.13） |
| Azerbaijan | 3355.87 （2490.91-4697.71） | 27.35 （20.45-37.71） | 4.34 （2.68-6.15） |  | 170.04 （162.07-177.80） | 0.31 （0.25-0.36） | 0.55 （-0.05-4.79） |  | 2254.95 （1851.50-2540.47） | 157.91 （146.69-169.16） | 0.6 （0.04-3.43） |
| Bangladesh | 8314.63 （3680.71-29140.41） | 5.05 （2.25-18.68） | 0.00 （0.00-0.00） |  | 358.51 （3.61-2262.55） | 0.23 （0.00-1.46） | 0.00  (0.00-0.00) |  | 21238.71 （1691.50-125468.43） | 13.25 （1.03-78.55） | 0.00  (0.00-0.00) |
| Equatorial Guinea | 71378.53 （44945.49-109715.80） | 6408.12 （4177.45-9505.96） | 28.49 （11.06-74.61） |  | 20722.24 （18852.90-23072.47） | 204.35（130.73-323.02） | 31.67 （13.66-68.35） |  | 121222.11 （75452.68-201408.50） | 10386.15 （6554.59-16650.60） | 27.11 （11.14-58.84） |
| Finland | 1361.99 （908.37-1913.06） | 20.19 （12.96-28.27） | 2.76 （1.87-3.66） |  | 22.48 （21.28-23.68） | 0.08 （0.08-0.09） | -0.67 （-0.69--0.65） |  | 329.79 （281.43-395.35） | 5.6 （4.86-6.62） | -0.58 （-0.64- -0.51） |
| Djibouti | 13334.83 （7387.76-23734.59） | 1137.32 （622.81-2015.58） | 72.54 （24.27-215.18） |  | 903.02 （647.69-1296.40） | 81.48 （58.40-116.40） | 483.64 （140.60-4339.21） |  | 45558.84 （31735.61-66528.60） | 3863.82 （2731.16-5520.77） | 294.72 （98.41-1240.92） |
| Syrian Arab Republic | 383.82 （276.07-589.62） | 2.67 （1.91-4.09） | 1.88 （1.45-2.47） |  | 46297.75 （40986.60-53461.46） | 0.11 （0.08-0.14） | 0.59 （0.25-1.05） |  | 867.06 （684.02-1125.33） | 1536.37 （1142.03-2005.13） | 0.58 （0.28-0.92） |
